# Assessing SimCLIM climate model accuracy in projecting Southern Levantine basin air temperature trends up to 2100

**DOI:** 10.1038/s41598-023-46286-7

**Published:** 2023-11-03

**Authors:** Nada M. Salama, Rongshuo Cai, Kareem Tonbol

**Affiliations:** 1 Oceanography Department, Faculty of Science, Alexandria University, Alexandria, Egypt; 2 Third Institute of Oceanography, Ministry of Natural Resources, Xiamen, China; 3 College of Maritime Transport and Technology, Arab Academy for Science, Technology and Maritime Transport, Alexandria, Egypt

**Keywords:** Climate sciences, Environmental sciences

## Abstract

This study evaluates the validity of forecasting air temperature ranges in 2100 using the SimCLIM climate projection model at spatial and temporal scales within the Southern Levantine basin. The model utilized historical air temperature data from 2000 to 2016, collected at seven southeastern Mediterranean stations, as well as 74 climate pattern ensembles integrated within SimCLIM. A combination of 40 global climate models (GCMs) and IPCC AR5 greenhouse gas emissions scenarios embedded in SimCLIM was employed to forecast mean, minimum, and maximum temperatures for 2100.The findings reveal that the average temperature increase in 2100, relative to the representative concentration pathways 2.6, 4.5, 6.0, and 8.5, will range between 0.8–1.17 °C, 1.48–2.0 °C, 2.1–3.8 °C, and 3.9–4.6 °C, respectively. Due to its acceptable accuracy, the SimCLIM model, incorporating 40 GCMs and 74 climate pattern ensembles, is highly recommended for forecasting future climate conditions. The model was evaluated using available temperature records in the study area, yielding a prediction percentage error of 2%, which strongly supports the use of SimCLIM.

## Introduction

Climate change has far-reaching and unlimited consequences. Worldwide negotiations and efforts have been initiated since 1990 to address the climate crisis, as reported by Guglyuvatyy. Controlling the increase in the Earth's temperature is a global challenge, primarily attributed to the release of carbon dioxide from the combustion of coal and fossil fuels, which is the leading cause of temperature rise^1^. There are global predictions of temperature increases, which are based on multiscale climate models that take into account regional variations^2,3^. According to the sixth assessment report published by Working Group I of the Intergovernmental Panel on Climate Change (IPCC), as mentioned by Lee et al.^4^, the average warming trend by 2100 may range from 1.1 to 5.4 °C. However, the coarse resolutions of global climate models and various regional variations introduce uncertainties into the outcomes of these General Circulation Models (GCMs)^5^. Therefore, to reduce uncertainties, there is a need to focus on regional modeling, utilizing high-resolution regional climate models.

Coastal communities face numerous hazards due to climate change, including sea-level rise and heatwaves^6^. Egypt’s Mediterranean coast is host to several large and captivating cities. Nevertheless, the majority of Egypt's coastal zones are inherently unstable and vulnerable to extensive activities^7^. As a result, it becomes imperative to focus on regional changes in climate factors, with air temperature ranking among the most crucial factors that require periodic study and monitoring. Utilizing numerical modeling techniques to investigate climate conditions yields more accurate results compared to other methodologies. This study employed the SimCLIM climate model, which integrates data from 40 General Circulation Models (GCMs) to analyze regional climate through downscaling techniques. Furthermore, SimCLIM utilizes 74 regional climate model patterns, which are then applied to site-specific data. Additionally, the pattern scaling technique has been incorporated into the downscaling mechanisms, enabling SimCLIM users to predict both short-term climate variability and long-term climate trends, in accordance with the Representative Concentration Pathways (RCPs) 8.5, 6.0, 4.5, and 2.6.

**Figure 1:**
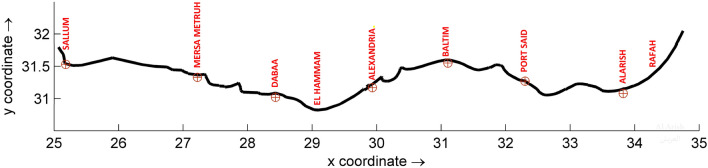
The Egyptian Mediterranean coast with the locations of the stations marked by red circles. This image was created using Delft Dashboard (https://publicwiki.deltares.nl/display/DDB/Delft+Dashboard).

This study focuses on analyzing air temperature trends in the Egyptian Mediterranean coastal area (see Fig. 1). Hasanean^8^ examined winter temperatures in Egypt from 1905 to 2000 and found them to be generally constant, with greater variability in the south compared to the north. However, most stations have experienced warming, especially in the last two decades, due to human activities and atmospheric changes. Notably, the North Atlantic Oscillation (NAO) and East Atlantic-West Russia (EAWR) indices have had a more significant impact on Egypt’s winter temperatures than El Niño or East Atlantic indexes.

Domroes and El-Tantawil^9^ analyzed site data from 1971 to 2000 and reported a positive temperature trend of 0.18 °C per decade in the westernmost coastal sector. In the Alexandria coastal sector, using observations from 1941 to 2000, they found a warming trend of 0.1 °C per decade, while the North Sinai coastal sector showed a negative warming trend of − 0.06 °C per decade. Hasanean and Basset^10^ demonstrated stable summer temperature patterns across Egypt based on data from 19 distinct locations. Nevertheless, temperatures have significantly increased over the past two decades, driven by human activity and shifts in meteorological conditions. Said et al.^11^ reported a substantial increase in the average annual air temperature in the Alexandria region from 1979 to 2011. During this period, temperatures rose by approximately 2.24 °C, representing an increase of roughly 0.6 °C per decade. Shaltout et al.^12^ used ERA-Interim data and global climate models to forecast the future climate of Egypt's Mediterranean coast (EMC). The statistics from 1979 to 2000 indicated rising air temperatures, decreasing precipitation, and declining sea-level pressure in the EMC. The most reliable model, CgCM 3.1, predicts increased warming, more frequent droughts, and a minor decrease in sea-level pressure for the EMC by the end of the century.

Kareem et al.^14^ analyzed hourly temperature observations from 2007 to 2016 along the Egyptian Mediterranean coast. The results showed a monthly temperature increase rate of 0.04 °C at 20 m above mean sea level (MSL) in the westernmost sector (Marsa Matruh), while the rate was 0.002 °C at 21.95 m above MSL in Alexandria's coastal region (Ras El-Teen). In the Nile Delta coastal sector, at an elevation of 26.6 m above MSL, there was a decline of 0.001 °C per month. Furthermore, along the North Sinai coastal sector, with an average elevation of 17 m above MSL, a positive temperature trend was observed. According to the World Bank^15^, Egypt’s climate is characterized as hot and dry, with milder winters and some coastal rain. Coastal temperatures range from 14 in winter to 30 °C in summer, while interior temperatures vary from 0 to 43 °C. Seasonal “khamsin” windstorms can significantly elevate national temperatures.

This study aims to investigate the long-term air temperature trends in the Egyptian Mediterranean coastal area until 2100, considering different Representative Concentration Pathways (RCPs). To achieve this goal, SimCLIM utilizes 40 GCM patterns on air temperature data derived from the International Cordex database at a 1 × 1 KM resolution. Additionally, 74 pattern ensembles, derived from regional climate models (RCMs), are applied to the measured site air temperature data. The geographic locations of the measuring stations are depicted in Fig. 1.

## Study area and data sources

### Study area

The study area encompasses the Mediterranean coastline of Egypt, situated between 25° E and 34.5° E longitude and 30° N and 33° N latitude, within the southern Levantine sub-basin. This coastal region spans approximately 1000 km, stretching from Rafah to Sallum, as depicted in Fig. 1. It is categorized based on its geological and physiographic characteristics into four distinct sectors: the North Sinai coastal sector, which extends from Rafah to Port Said (180 km); the Nile Delta coastal sector, which stretches from Port Said to Alexandria (240 km); the Alexandria coastal sector, which ranges from Abu Qir Headland to Hammam (70 km); and the westernmost sector of the Egyptian Mediterranean coast, which extends for about 550 km from Hammam to Sallum. These divisions and geographic boundaries have been reported by Frihy and El-Sayed^16^ and Abayazid et al.^17^.

### Historical site air temperature data

Air temperature data were recorded at seven locations along the Egyptian Mediterranean coast. Site-specific data were sourced from the Global Historical Climatology Network (GHCN) daily dataset, which is maintained by the National Oceanic and Atmospheric Administration's National Climate Data Center. SimCLIM center acquired these data, as indicated in Table 1. The dataset covers the period from 1957 to 2016, with a focus on the years 2000 to 2016 for the analysis.

**Table 1:** The air temperature historical site data (HGCN) for seven locations along the Egyptian Mediterranean coastal area supported by SimCLIM center, from (2000:2016).

Dataset		Source	Temporal resolution (h)	Geographic position	
Air temp	St.1	Salloum Plateau	24	31.53 °N	25.18 °E
	St.2	Mersa Metruh	24	31.33 °N	27.22 °E
	St.3	Dabaa	24	31.02 °N	28.43 °E
	St.4	Alexandria/Nouzha	24	31.17 °N	29.93 °E
	St.5	Baltim	24	31.55 °N	31.1 °E
	St.6	Port Said	24	31.27 °N	32.3 °E
	St.7	El Arish/Agrimet	24	31.08 °N	33.82 °E

### Cordex international database

The COordinated Regional Climate Downscaling EXperiment (CORDEX) is a framework supported by the World Climate Research Program (WCRP) that generates datasets of regional climate predictions for all continents worldwide.

## Methods

### SimCLIM model

The computer-based modeling system, SimCLIM 4.x for Desktop, is employed to study the impact of climatic variability and change over time and space. It offers a wide range of applications, including describing baseline climates, examining current climate extremes and variability, evaluating present and future hazards, researching adaptation strategies, inventing climate and sea-level rise scenarios, projecting sectoral consequences of climate change and sea-level rise, conducting sensitivity assessments, performing integrated impact studies, and assessing risks and uncertainties. Furthermore, it utilizes 40 General Circulation Models (GCMs), including ACCESS1.3, ACCESS1.0, BCC-CSM1-1, BCC-CSM1-1-m, BNU-ESM, CanESM2, CCSM4, CESM1-BGC, CESM1-CAM5, CMCC-CM, CMCC-CMS, CNRM-CM5, CSIRO-Mk3-6-0, EC-EARTH, FGOALS-g2, FGOALS-s2, GFDL-CM3, GFDL-ESM2G, GFDL-ESM2M, GISS-E2-H, GISS-E2-H-CC, GISS-E2-R, GISS-E2-R-CC, HADCM3, HadGEM2-AO, HadGEM2-CC, etc.

### Historical site data analysis

The air temperature dataset was analyzed by spatially comparing the monthly minimum, mean, and maximum temperature values across the study area during the base period from 2000 to 2016. The study area spans from Sallum (referred to as St. 1) in the westernmost sector to El Arish (referred to as St. 7) in the North Sinai sector.

### Downscaling using SimCLIM and forecasting long-term temperature trends

#### Downscaling from GCMs

Down-scaling mechanisms, including the pattern scaling technique, were employed with the SimCLIM model to process temperature data obtained from the CORDEX database and projected onto specific geographical locations. These selected locations correspond to the historical site data. Furthermore, air temperature forecasts will be generated from the base period (2000–2016) up to 2100, employing simple and direct descriptive statistics (minimum, mean, and maximum), with the selected Representative Concentration Pathways (RCPs) being 2.6, 4.5, 6.0, and 8.5.

As reported by Mitchell^18^, the pattern scaling technique depends on Eq. (1) where $({V}^{*})$ is the anomaly in a variable $(V)$ for a particular box domain $(i)$, during period year $(y)$ or months/season $(j)$ for a selected forcing scenario $(x)$, the annual global anomaly of Tmean, which can be calculated by modelling the chosen forcing scenario is the scalar quantity $(S)$, the response value at each box domain node is the scalar quantity $(z)$, and the response pattern is given by $({V}{\prime})$.

1 $${V}_{xijy}^{*}={S}_{xy} . {V}_{zij}{\prime}$$

#### Application of SimCLIM climate pattern on the historical site data

Climate models employ intricate patterns to simulate various components of the Earth's climate system, including temperature, precipitation, air circulation, and more. These patterns are constructed using mathematical formulae and scientific laws that govern the behavior of the Earth's atmosphere, oceans, land surfaces, and ice. In SimCLIM, specific climate model patterns will be applied to historical site data to illustrate temperature changes in the context of the Representative Concentration Pathways (RCPs) up to the year 2100, considering the base period from 2000 to 2016.Producing regional climate change patterns can be achieved through various approaches, such as statistical or dynamical downscaling. However, pattern scaling offers certain advantages over other techniques. Therefore, in this section, pattern scaling is also employed in the analysis. Some examples of the patterns used include CANESM2, CCSM4, CESM1-BGC, CESM1-CAM5, CMCC-CM, CMCC-CMS, CMCC-CM5, etc.

#### Model evaluation

Climate model evaluation is a crucial step in climate research. It assesses the efficacy of numerical climate models in simulating past, present, and future climatic conditions. This evaluation procedure aims to gauge how effectively these models reproduce observable climatic features, understand their limitations, and improve their accuracy. The simplest approach to model evaluation involves comparing model outputs with observational data and analyzing any discrepancies. Such comparisons require an understanding of the inherent flaws and uncertainties in the observational data, as reported by Flato et al.^19^ and Randall et al.^20^. A more sophisticated evaluation method involves selecting a different future period from the one initially examined while using the same evaluation techniques. In this study, the SimCLIM model was assessed by calculating the percentage prediction error between two sets of outputs projected for the year 2040 based on two simulation scenarios: RCPs 2.6 and 8.5. The first set of outputs consists of downscaled data from 40 GCMs generated by SimCLIM, while the second set is derived from a pattern scaling method embedded in pattern ensembles and applied to SimCLIM measured dataset. The Port Said station was specifically selected for this evaluation. Before initiating the evaluation process, the mean temperature (Tmean) data from SimCLIM, originally sourced from GHCN, were compared with another set of Tmean data for the same period (2007–2016), as published in Kareem et al.^14^, Table 2. It should be noted that both stations are located 2 m above sea level and under similar conditions. They are 4.5 km apart, each owned by a different institution. Descriptive statistics were applied to the raw data to ensure its accuracy and compatibility, and it was found that the Port Said station is the best station to undergo the evaluation procedure.

**Table 2:** Comparison between the mean temperatures of air measured in °C, (Tmean) derived from the Global Historical Climatology Network (GHCN), and (Tmean) published in the work of Kareem et al.^14^ at Port Said*.*

Year/Tmean	2007	2008	2009	2010	2011	2012	2013	2014	2015	2016
GHCN	21.58	21.87	21.52	22.54	21.43	21.48	21.63	NAN	21.77	22.01
Published	21.7	22.1	22	22.9	21.8	22	21.8	22.2	22	22

## Results

SimCLIM climate software presents results in two formats: numerically, with various options such as graphs, or in numerical form within CSV files. Additionally, results can be illustrated using maps with different colors. Figures 2, 3, 4, 5, 6, 7, 8, 9, 10, 11, 12, 13, 14, 15, 16, 17, 18, 19, 20, 21, 22, 23, 24, 25, 26, 27, 28, 29, 30, 31, 32, 33, 34, 35 represent the outputs of SimCLIM, with the graphs displaying data extracted in the form of numbers from CSV files and manipulated using Microsoft Excel. The maps serve as illustrative outcomes of the work and are produced using SimCLIM as a part of this study.

**Figure 2:**
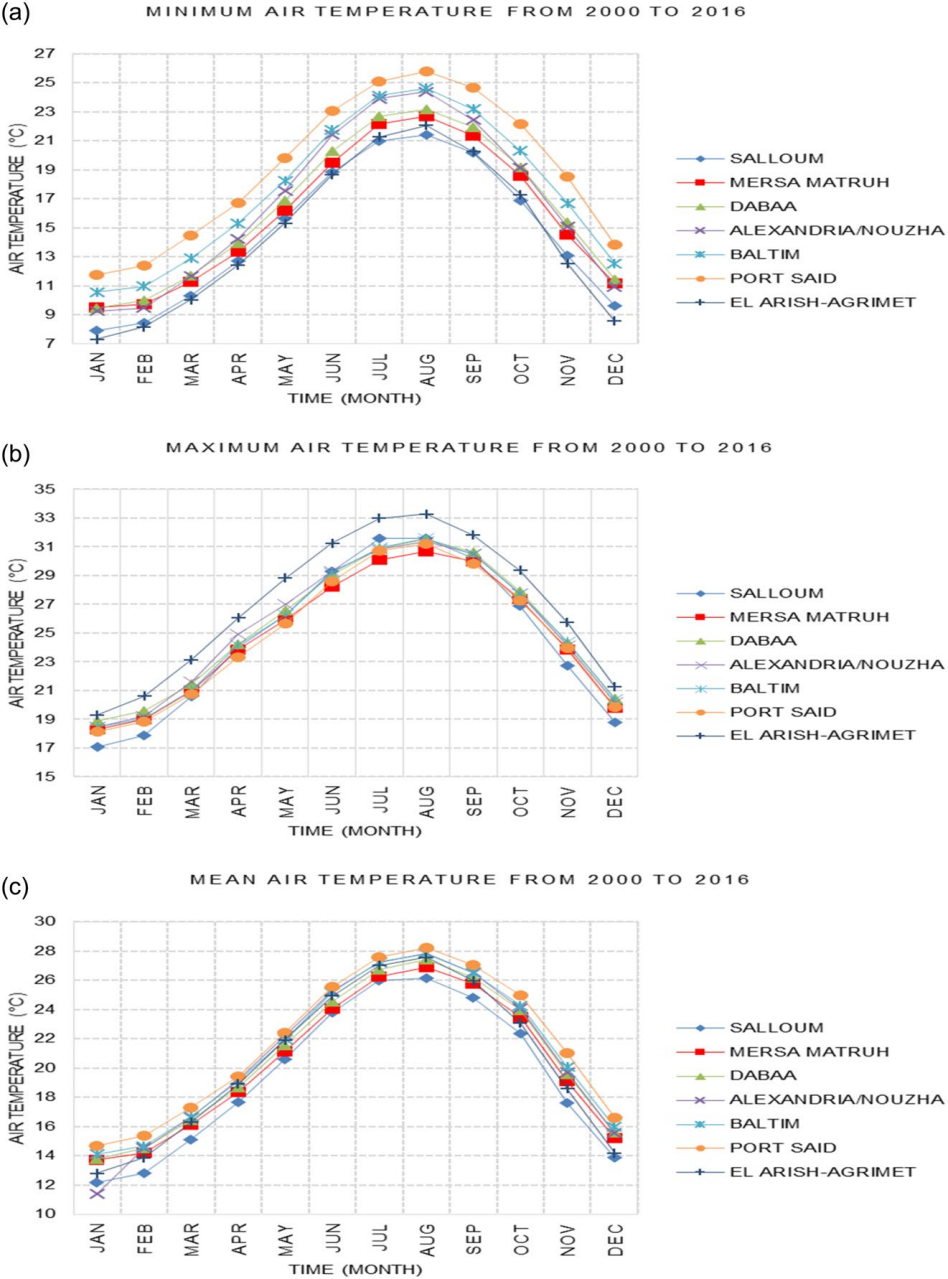
Monthly averages of the air temperature historical site data between 2000 and 2016. These graphs were created using Excel in the Microsoft Office Professional Plus 2016 package.

**Figure 3:**
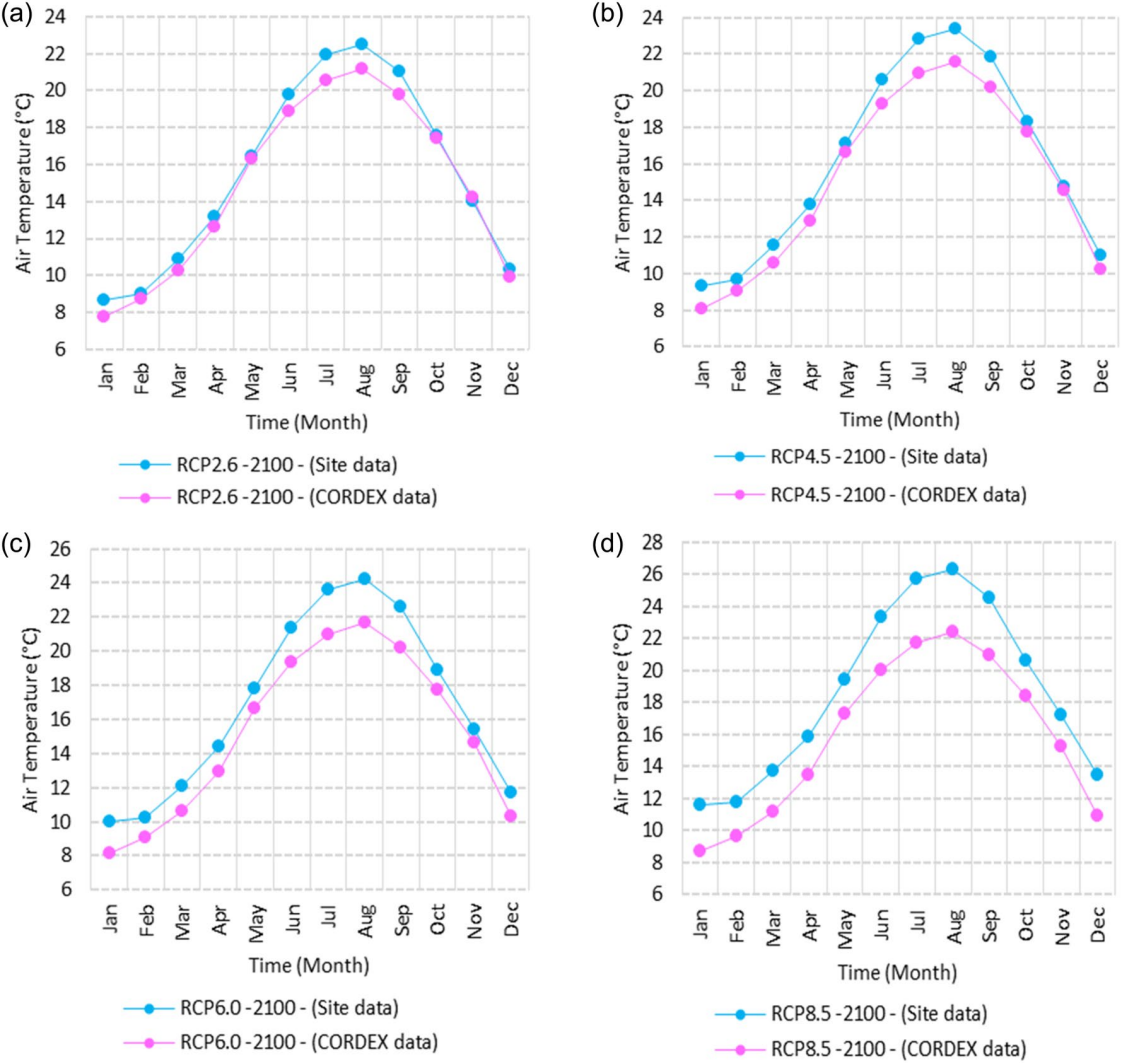
Averages of monthly minimum air temperatures according to the RCPs at Sallum. These graphs were created using Excel in the Microsoft Office Professional Plus 2016 package.

**Figure 4:**
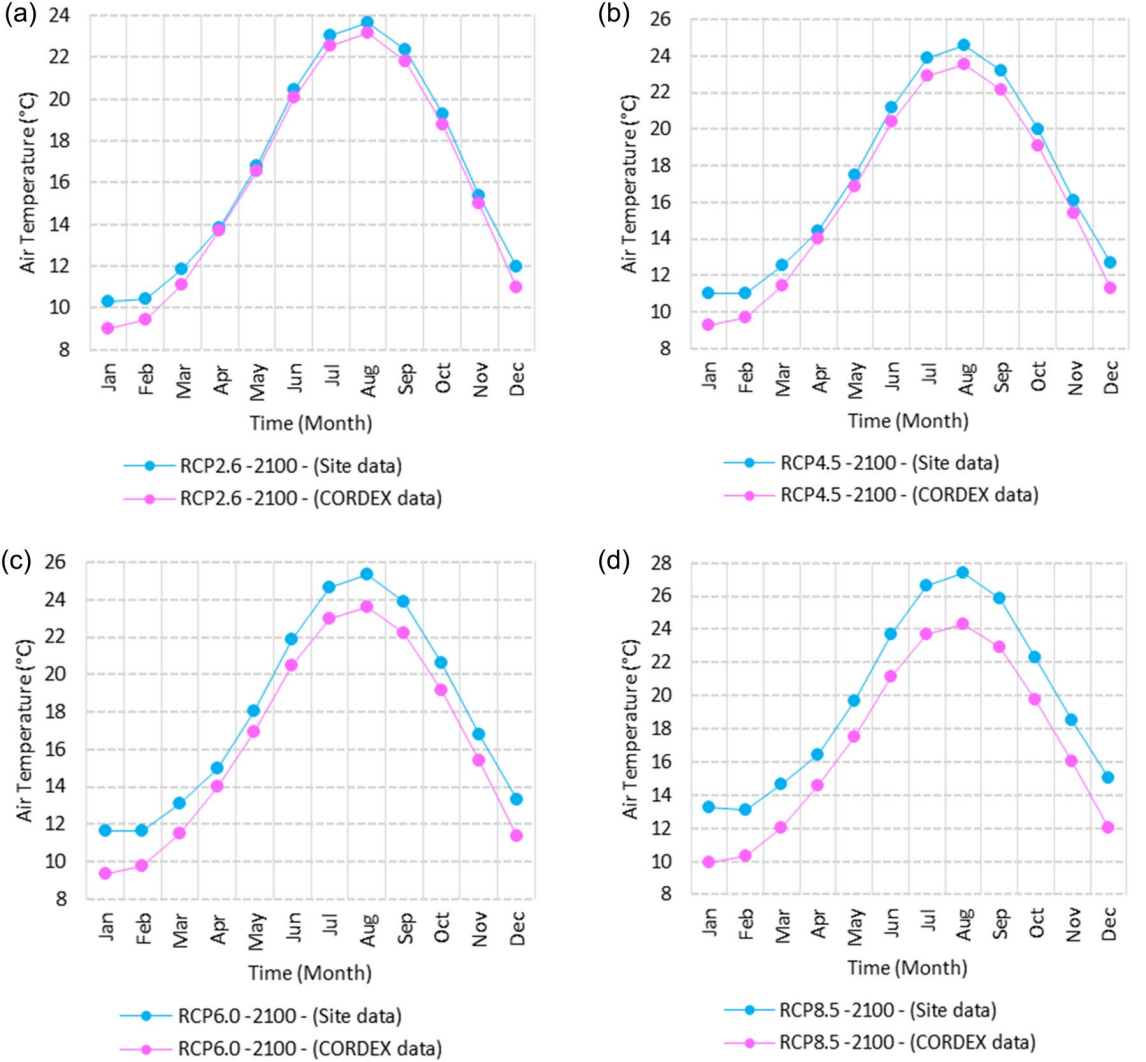
Averages of monthly minimum air temperatures according to the RCPs at Mersah Matruh. These graphs were created using Excel in the Microsoft Office Professional Plus 2016 package.

**Figure 5:**
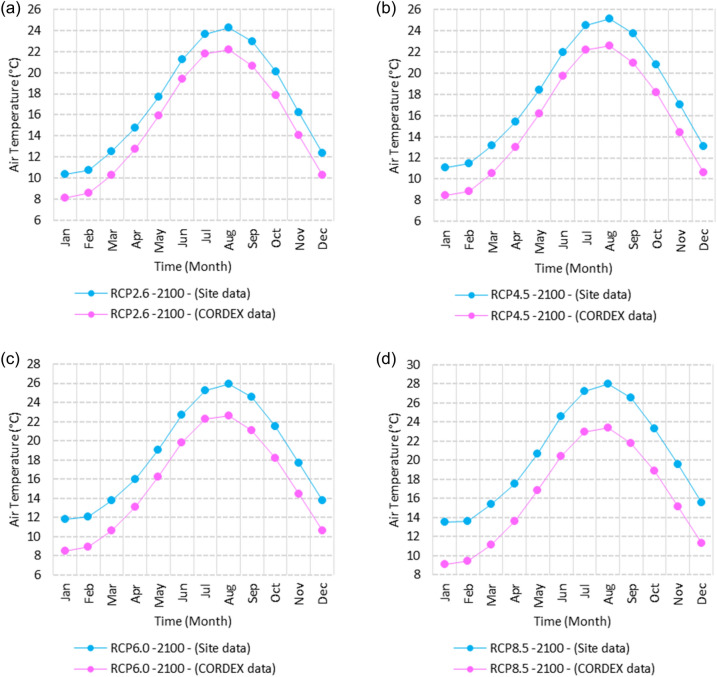
Averages of monthly minimum air temperatures according to the RCPs at Dabaa. These graphs were created using Excel in the Microsoft Office Professional Plus 2016 package.

**Figure 6:**
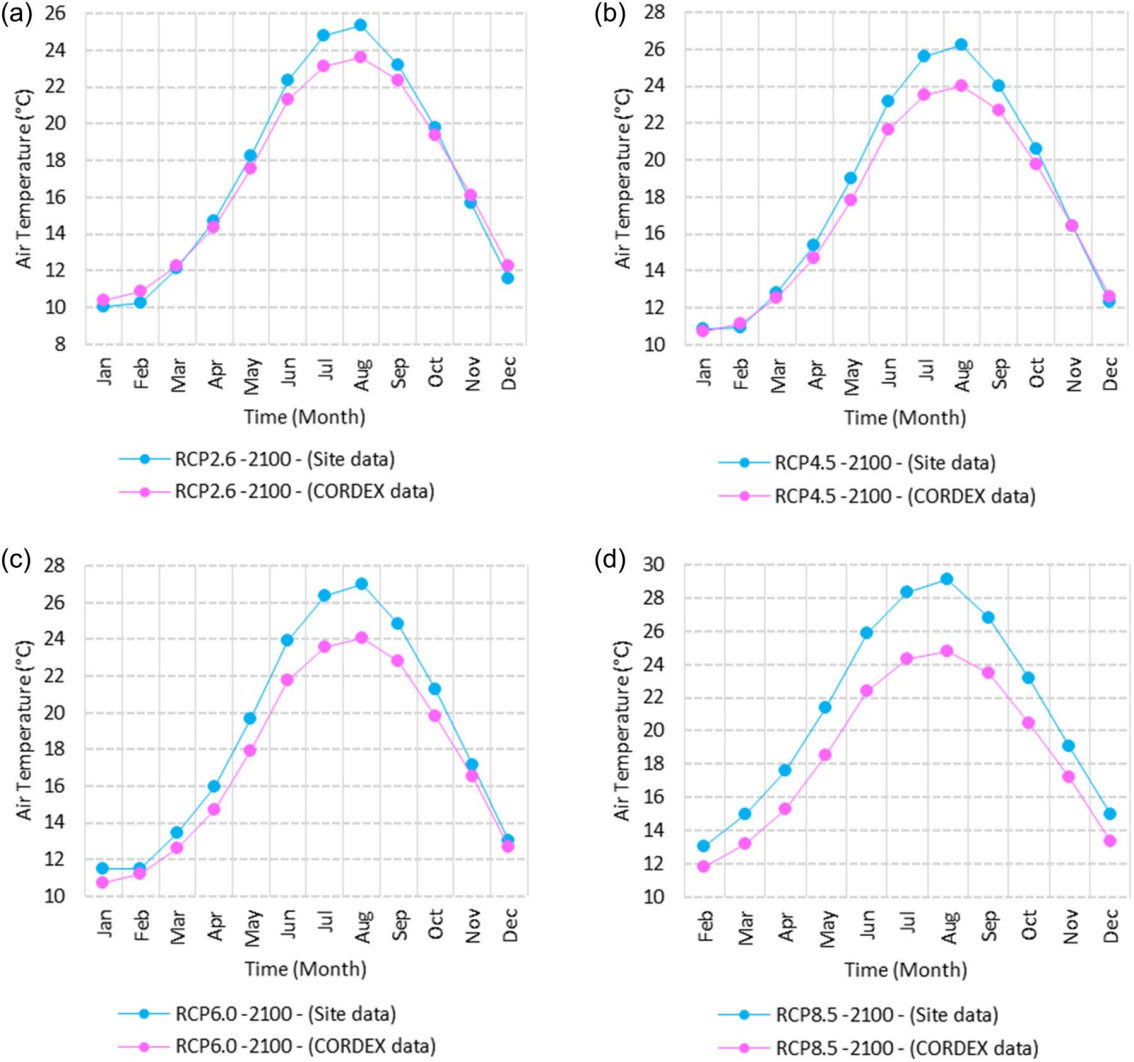
Averages of monthly minimum air temperatures according to the RCPs at Alexandria. These graphs were created using Excel in the Microsoft Office Professional Plus 2016 package.

**Figure 7:**
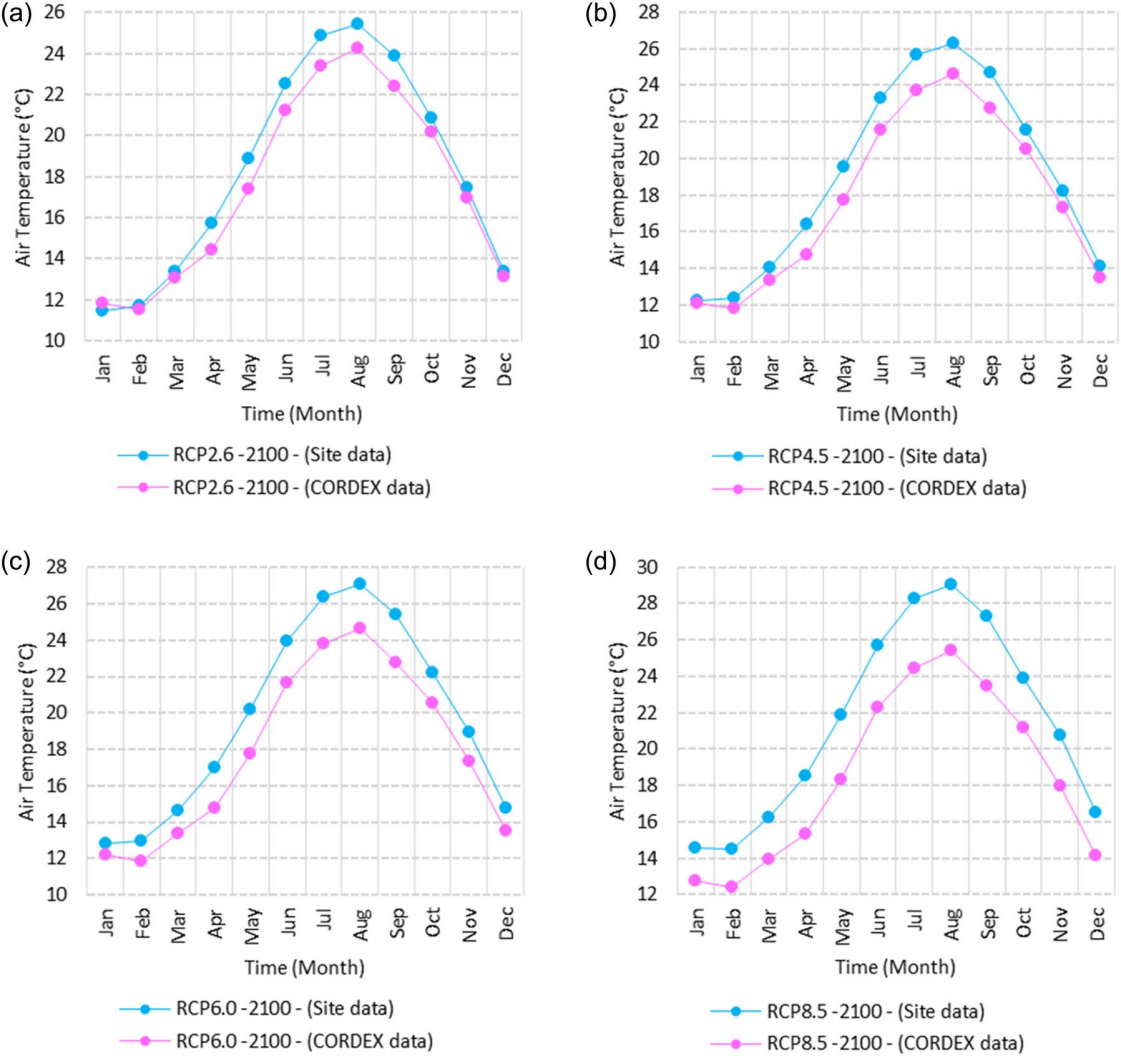
Averages of monthly minimum air temperatures according to the RCPs at Baltim. These graphs were created using Excel in the Microsoft Office Professional Plus 2016 package.

**Figure 8:**
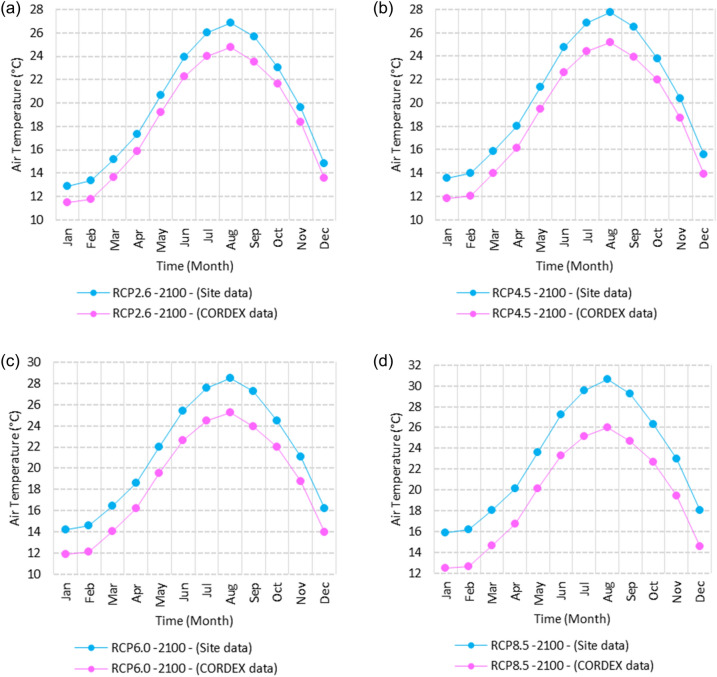
Averages of monthly minimum air temperatures according to the RCPs at Port Said. These graphs were created using Excel in the Microsoft Office Professional Plus 2016 package.

**Figure 9:**
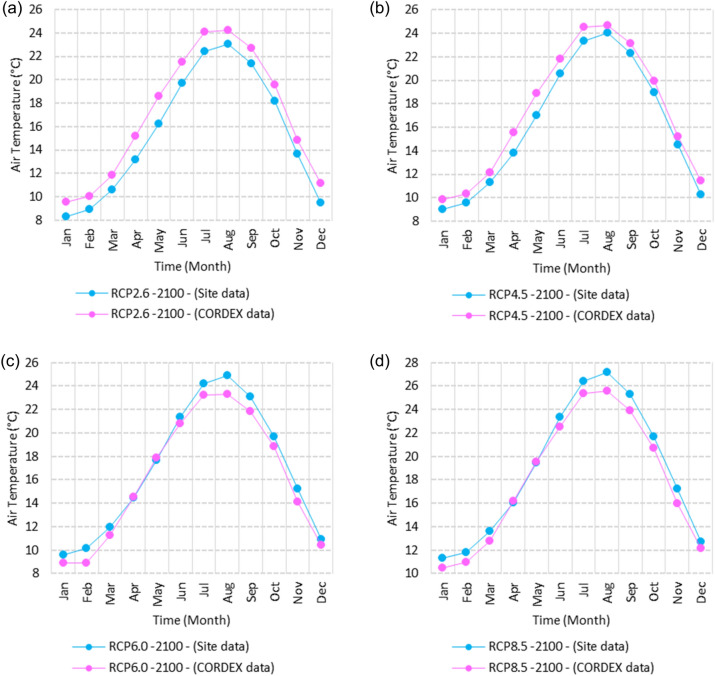
Averages of monthly minimum air temperatures according to the RCPs at Al Arish. These graphs were created using Excel in the Microsoft Office Professional Plus 2016 package.

**Figure 10:**
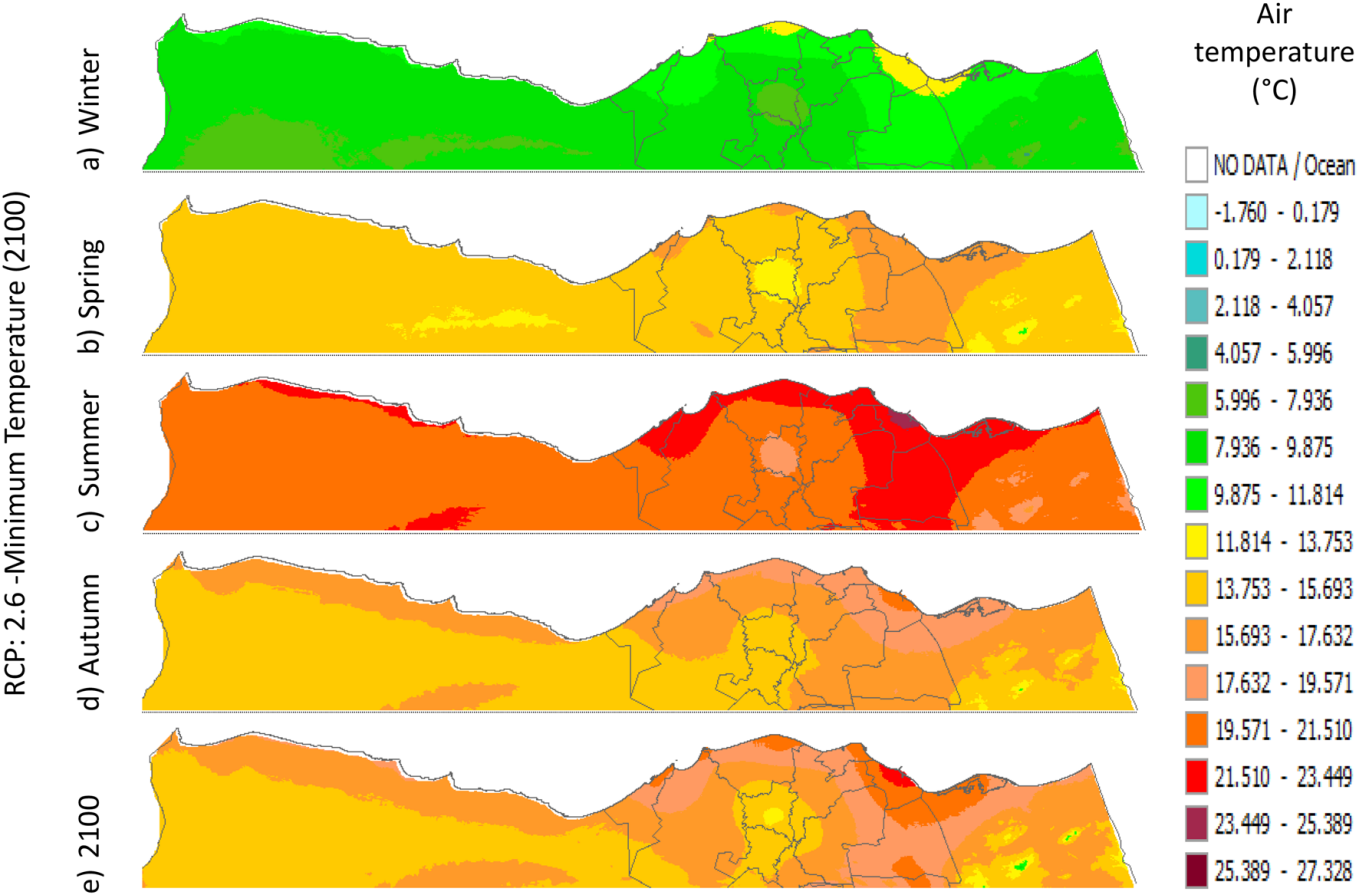
Distributions of the Projected seasonal and annual minimum air temperatures during 2100, according to RCP 2.6, along the Egyptian Mediterranean coast. The illustration was generated using SimCLIM v4.x for Desktop (SimCLIM AR5 (climsystems.com)).

**Figure 11:**
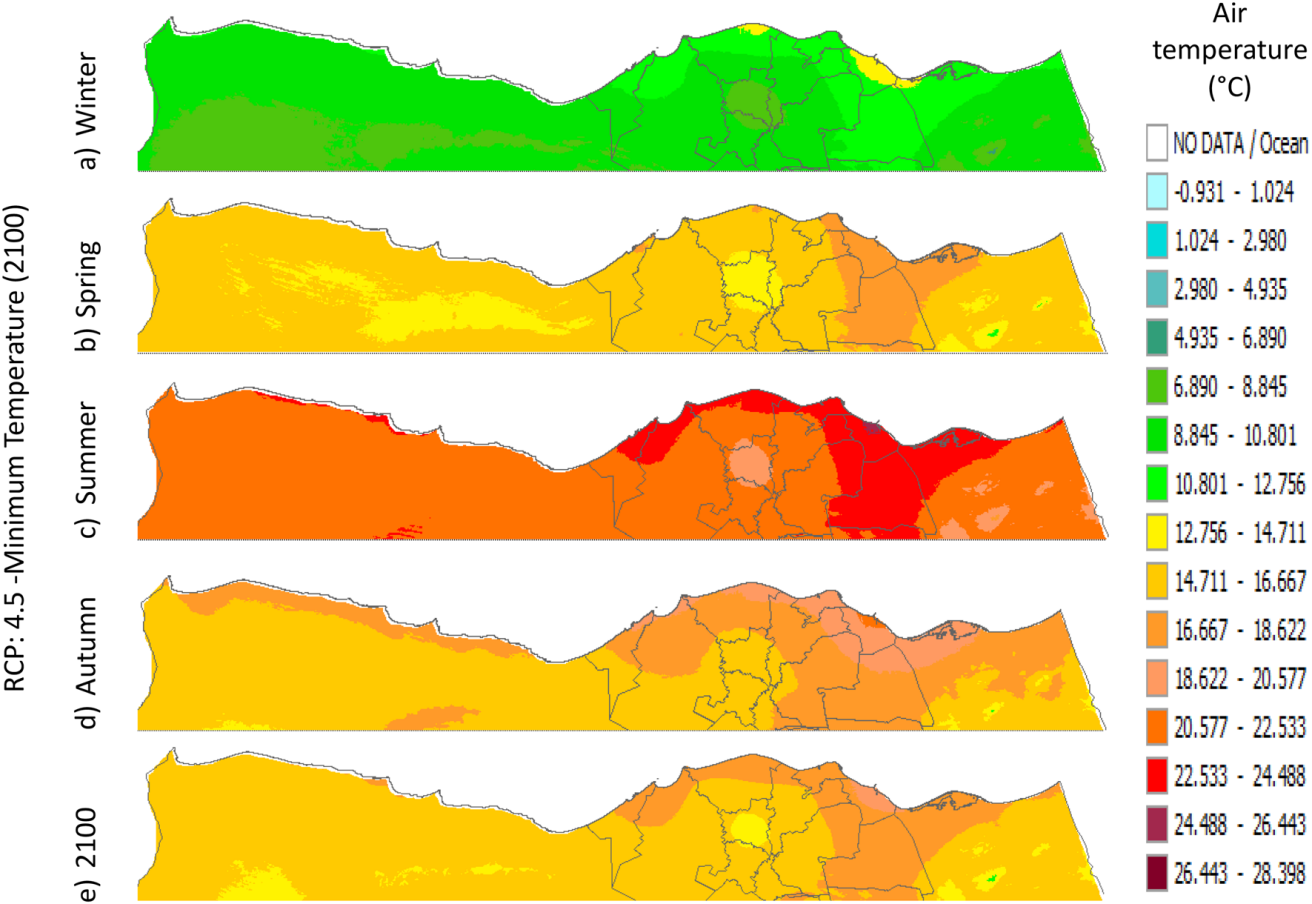
Distributions of the Projected seasonal and annual minimum air temperatures during 2100, according to RCP 4.5, along the Egyptian Mediterranean coast. The illustration was generated using SimCLIM v4.x for Desktop (SimCLIM AR5 (climsystems.com)).

**Figure 12:**
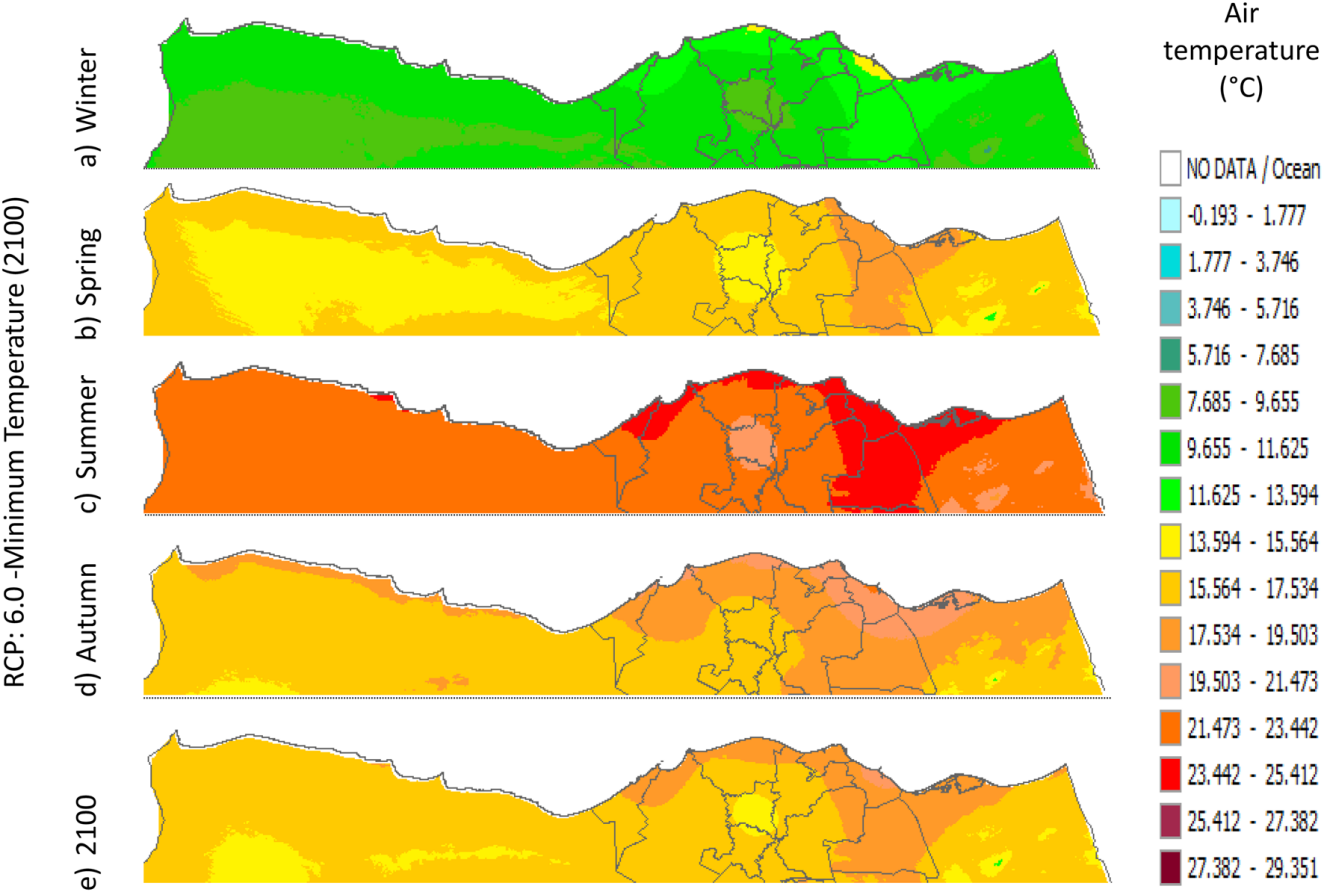
Distributions of the Projected seasonal and annual minimum air temperatures during 2100, according to RCP 6.0, along the Egyptian Mediterranean coast. The illustration was generated using SimCLIM v4.x for Desktop (SimCLIM AR5 (climsystems.com)).

**Figure 13:**
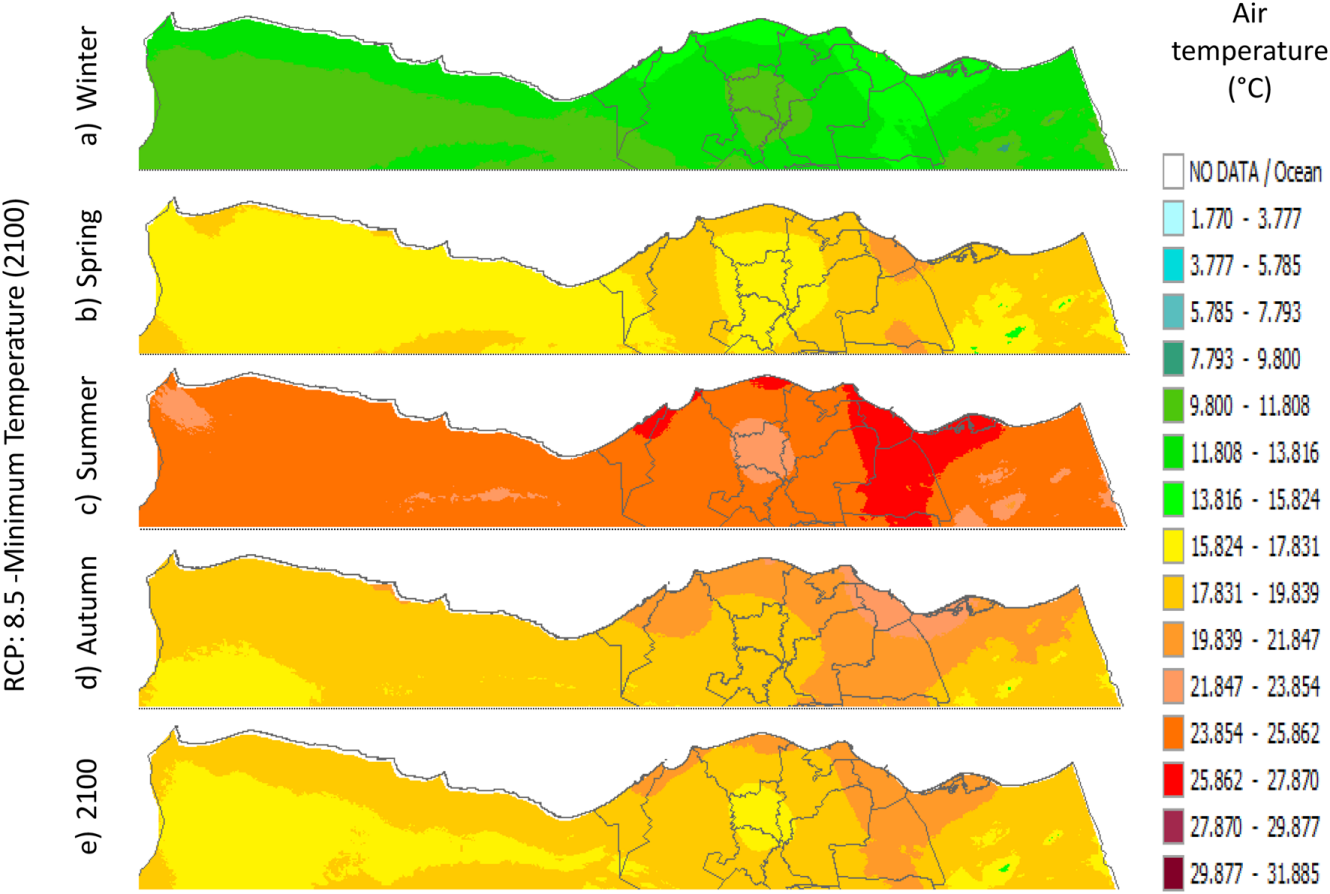
Distributions of the Projected seasonal and annual minimum air temperatures during 2100, according to RCP 8.5, along the Egyptian Mediterranean coast. The illustration was generated using SimCLIM v4.x for Desktop (SimCLIM AR5 (climsystems.com)).

**Figure 14:**
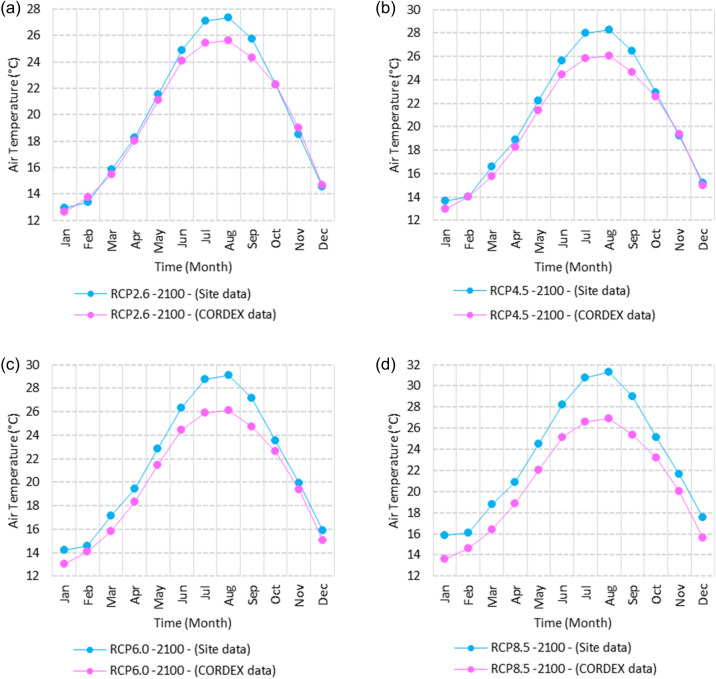
Averages of monthly mean air temperatures according to the RCPs at Sallum. These graphs were created using Excel in the Microsoft Office Professional Plus 2016 package.

**Figure 15:**
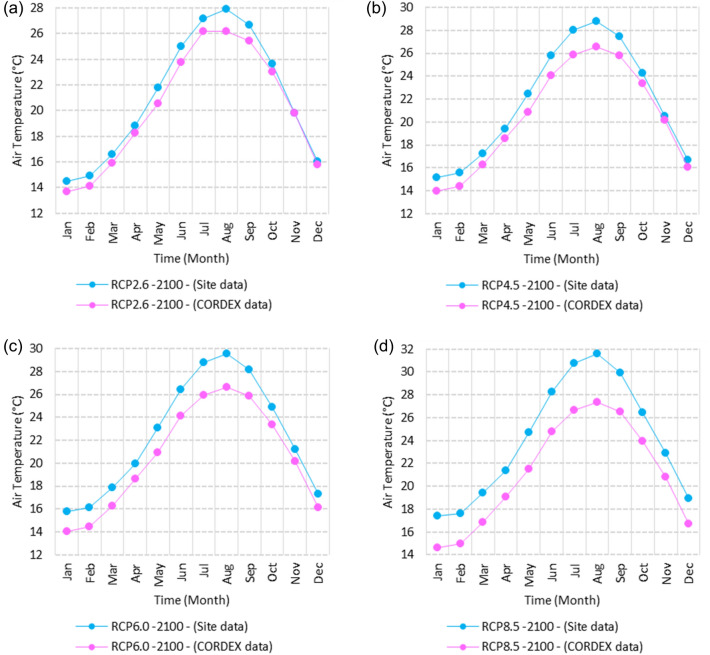
Averages of monthly mean air temperatures according to the RCPs at Mersah Matruh. These graphs were created using Excel in the Microsoft Office Professional Plus 2016 package.

**Figure 16:**
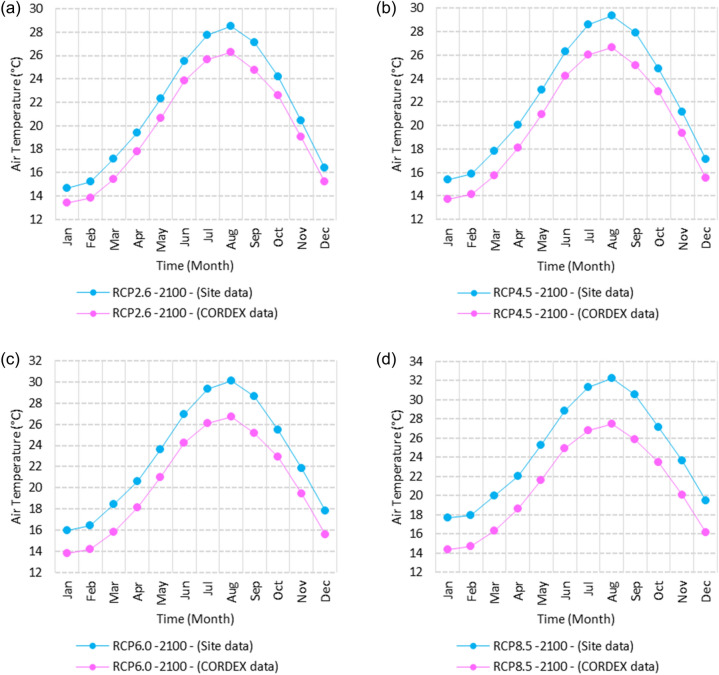
Averages of monthly mean air temperatures according to the RCPs at Dabaa. These graphs were created using Excel in the Microsoft Office Professional Plus 2016 package.

**Figure 17:**
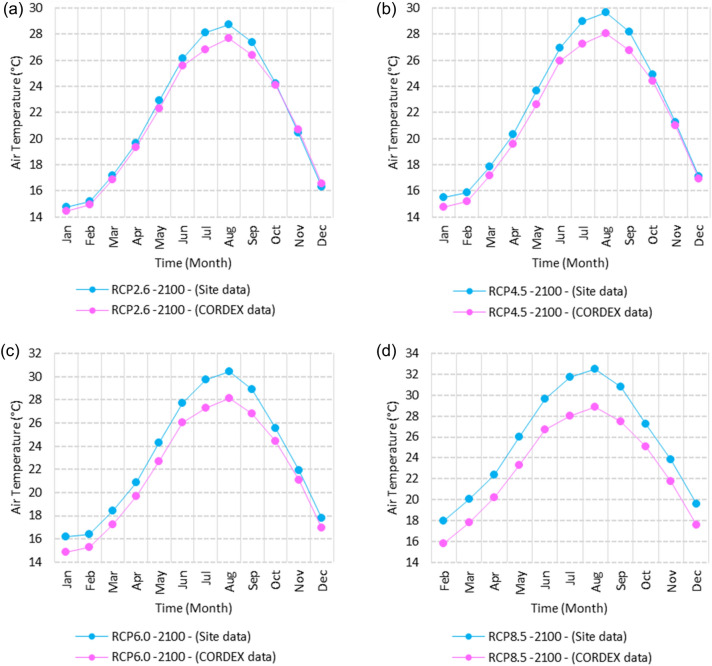
Averages of monthly mean air temperatures according to the RCPs at Alexandria. These graphs were created using Excel in the Microsoft Office Professional Plus 2016 package.

**Figure 18:**
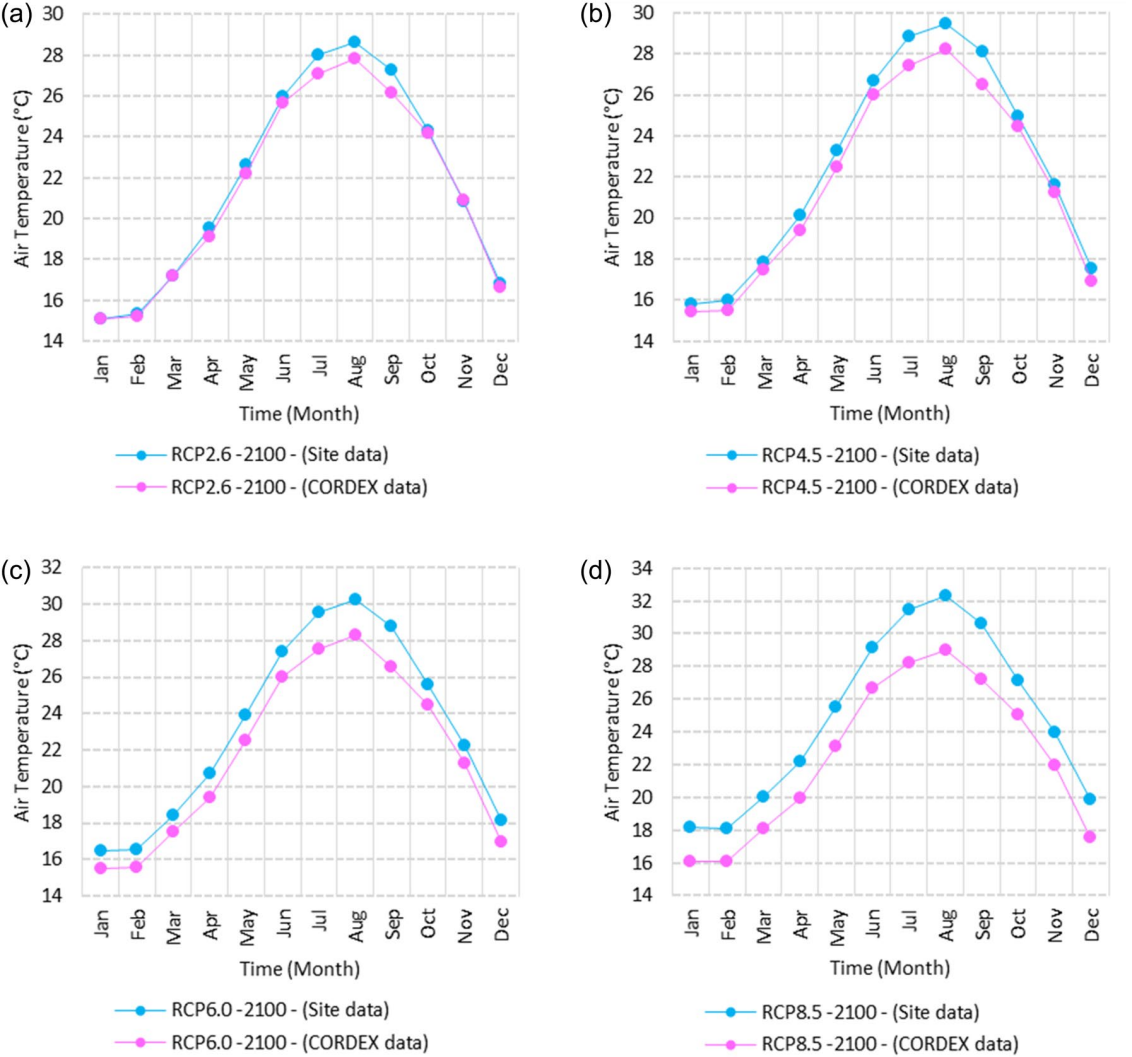
Averages of monthly mean air temperatures according to the RCPs at Baltim. These graphs were created using Excel in the Microsoft Office Professional Plus 2016 package.

**Figure 19:**
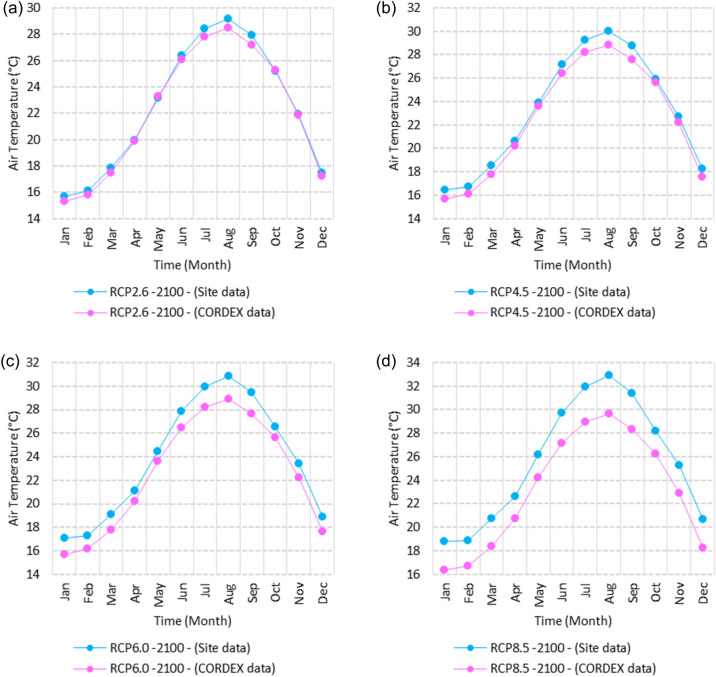
Averages of monthly mean air temperatures according to the RCPs at Port Said. These graphs were created using Excel in the Microsoft Office Professional Plus 2016 package.

**Figure 20:**
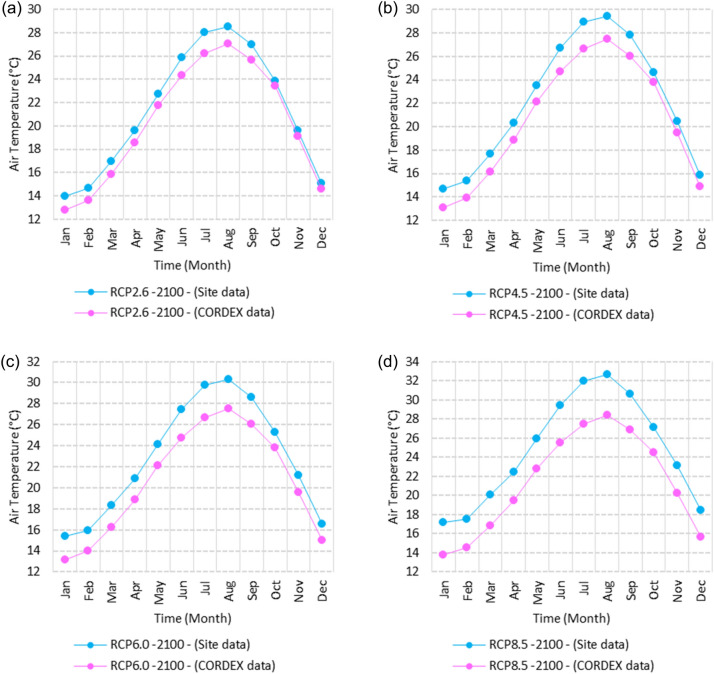
Averages of monthly mean air temperatures according to the RCPs at Al Arish. These graphs were created using Excel in the Microsoft Office Professional Plus 2016 package.

**Figure 21:**
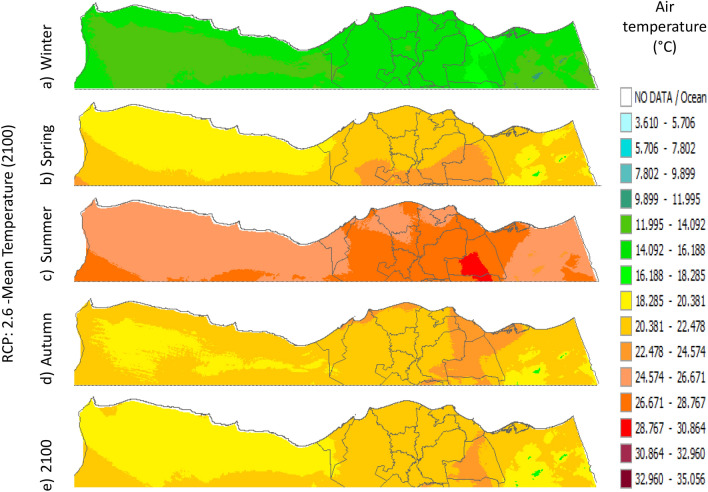
Distributions of the Projected seasonal and annual mean air temperatures during 2100, according to RCP 2.6, along the Egyptian Mediterranean coast. The illustration was generated using SimCLIM v4.x for Desktop (SimCLIM AR5 (climsystems.com)).

**Figure 22:**
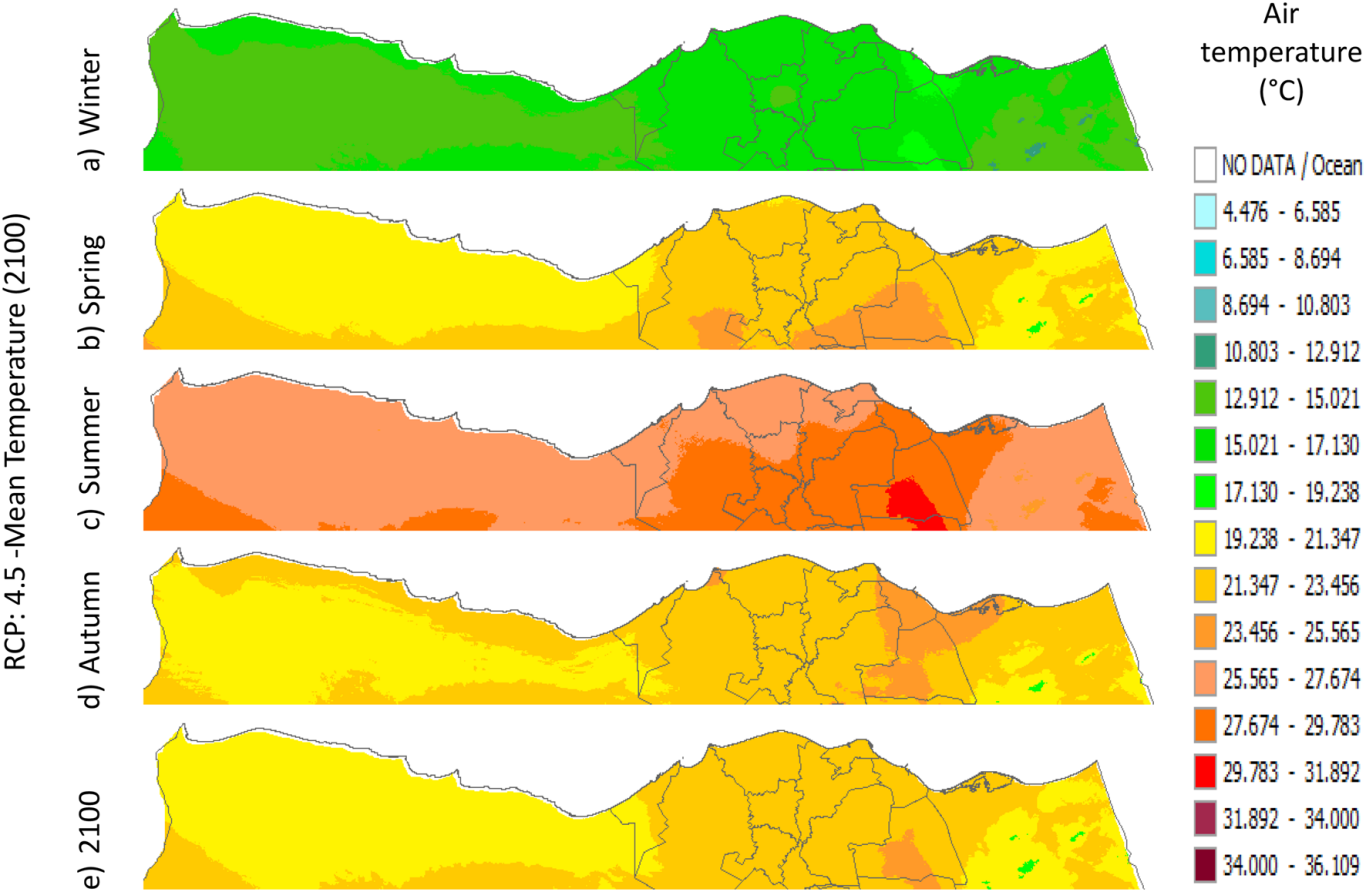
Distributions of the Projected seasonal and annual mean air temperatures during 2100, according to RCP 4.5, along the Egyptian Mediterranean coast. The illustration was generated using SimCLIM v4.x for Desktop (SimCLIM AR5 (climsystems.com)).

**Figure 23:**
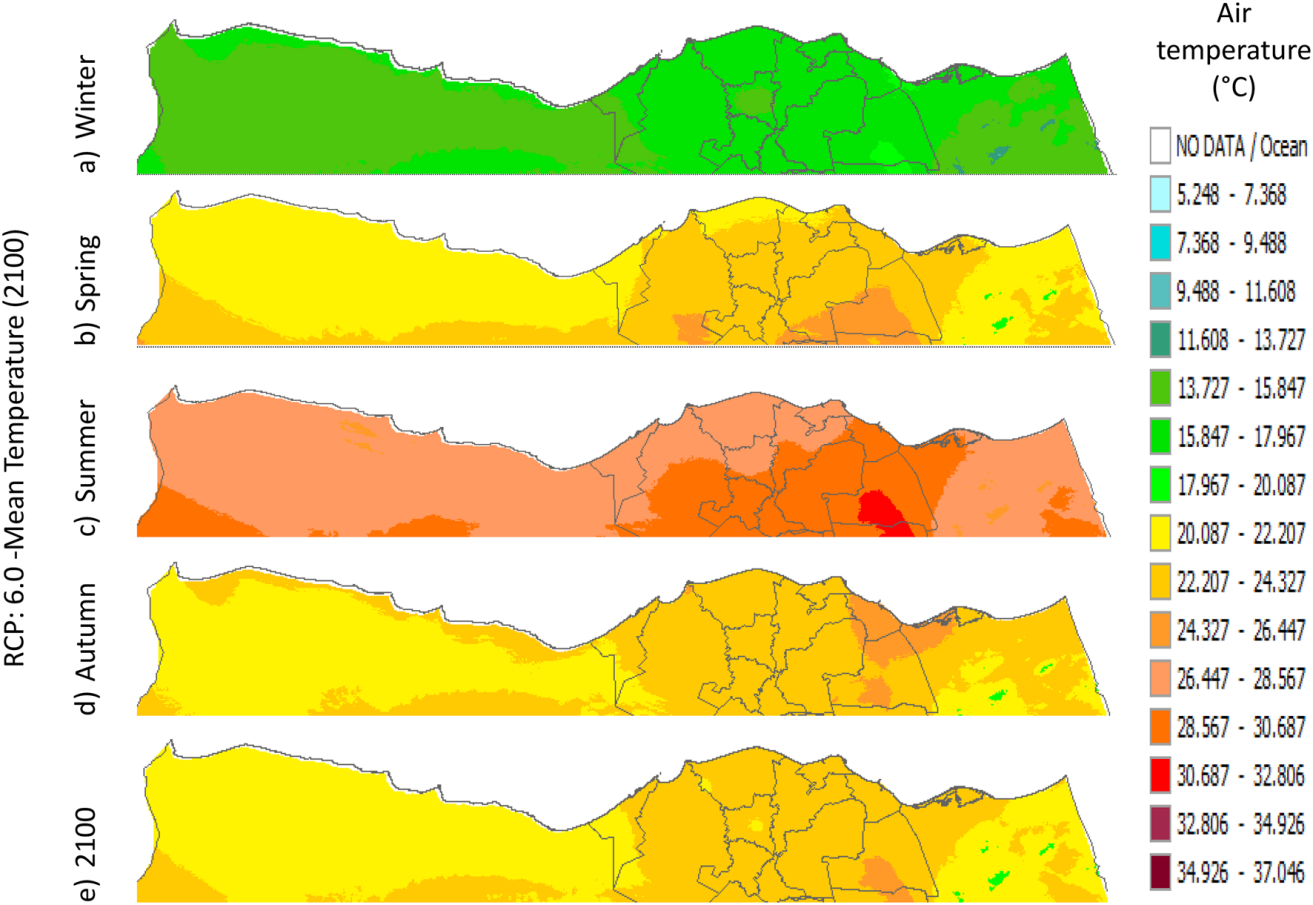
Distributions of the Projected seasonal and annual mean air temperatures during 2100, according to RCP 6.0, along the Egyptian Mediterranean coast. The illustration was generated using SimCLIM v4.x for Desktop (SimCLIM AR5 (climsystems.com)).

**Figure 24:**
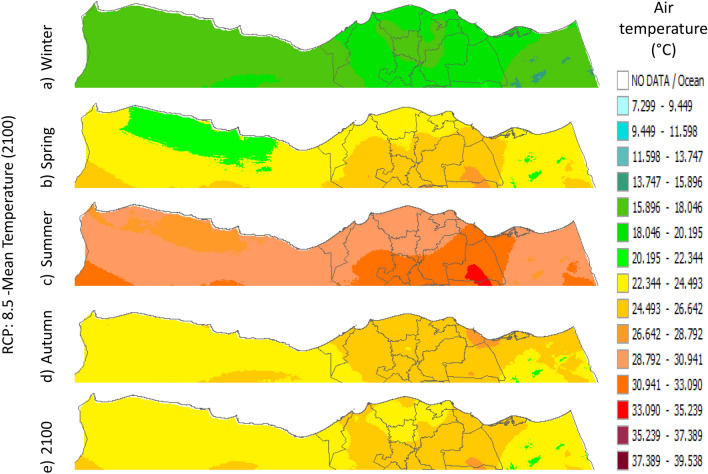
Distributions of the Projected seasonal and annual mean air temperatures during 2100, according to RCP 8.5, along the Egyptian Mediterranean coast. The illustration was generated using SimCLIM v4.x for Desktop (SimCLIM AR5 (climsystems.com)).

**Figure 25:**
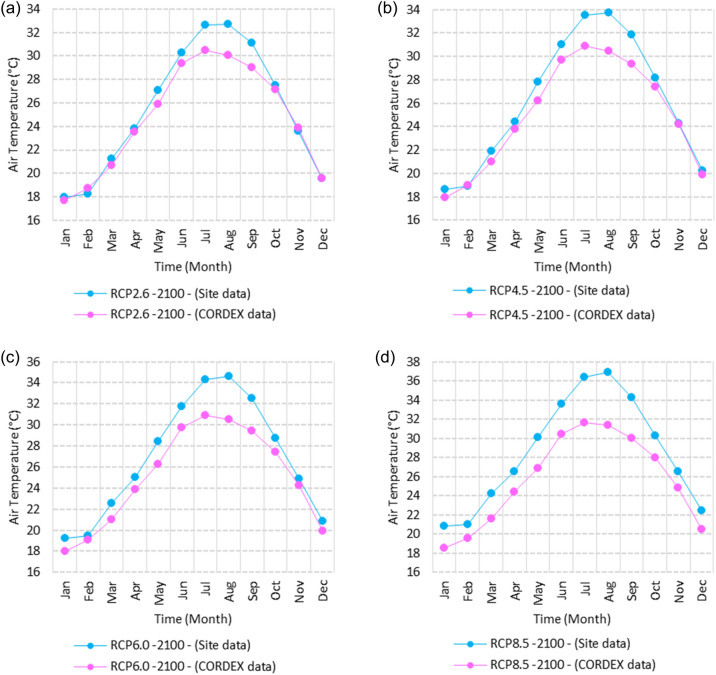
Averages of monthly maximum air temperatures according to the RCPs at Sallum. These graphs were created using Excel in the Microsoft Office Professional Plus 2016 package.

**Figure 26:**
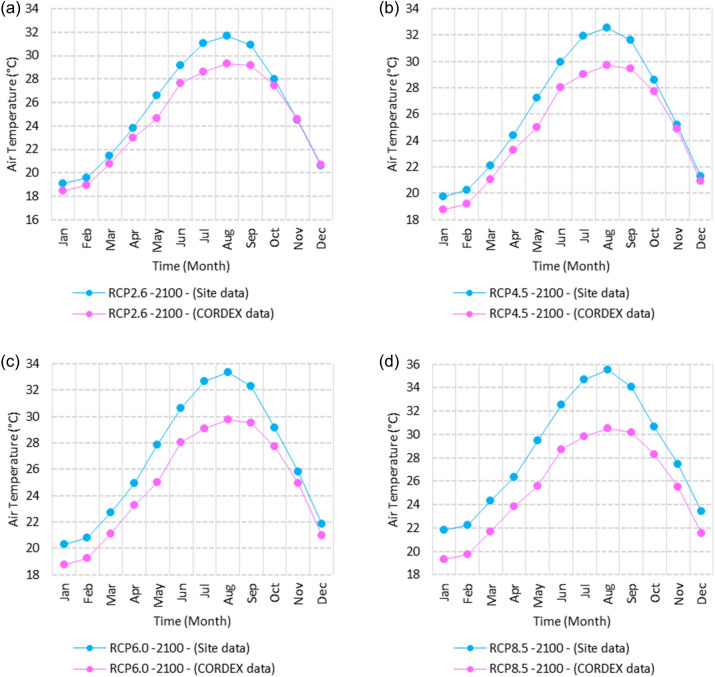
Averages of monthly maximum air temperatures according to the RCPs at Mersah Matruh. These graphs were created using Excel in the Microsoft Office Professional Plus 2016 package.

**Figure 27:**
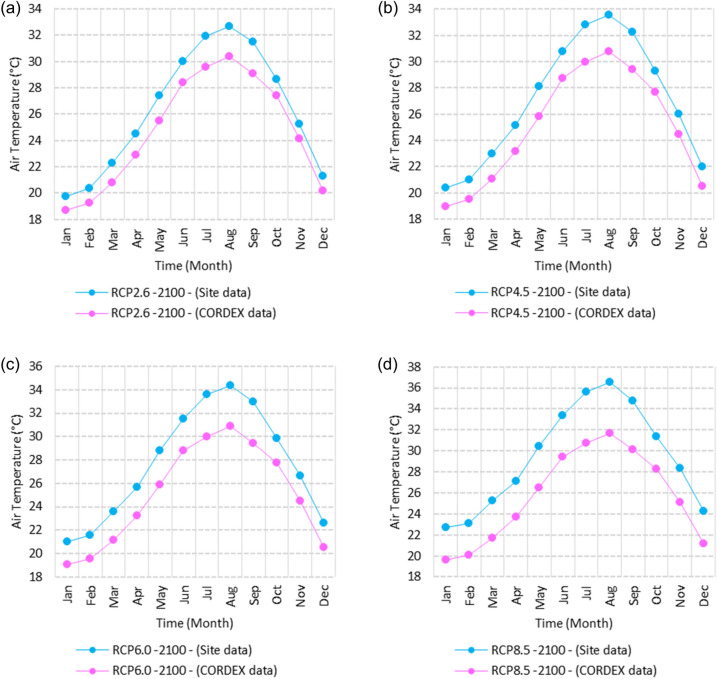
Averages of monthly maximum air temperatures according to the RCPs at Dabaa. These graphs were created using Excel in the Microsoft Office Professional Plus 2016 package.

**Figure 28:**
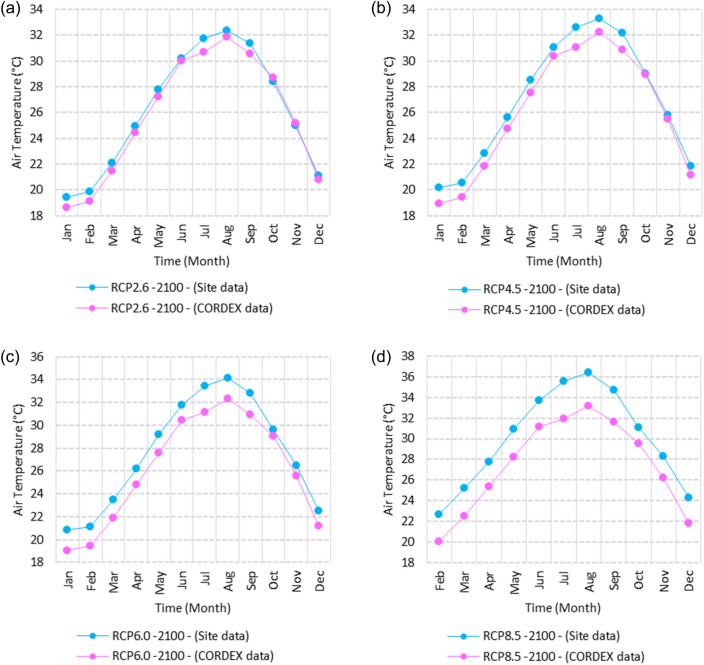
Averages of monthly maximum air temperatures according to the RCPs at Alexandria. These graphs were created using Excel in the Microsoft Office Professional Plus 2016 package.

**Figure 29:**
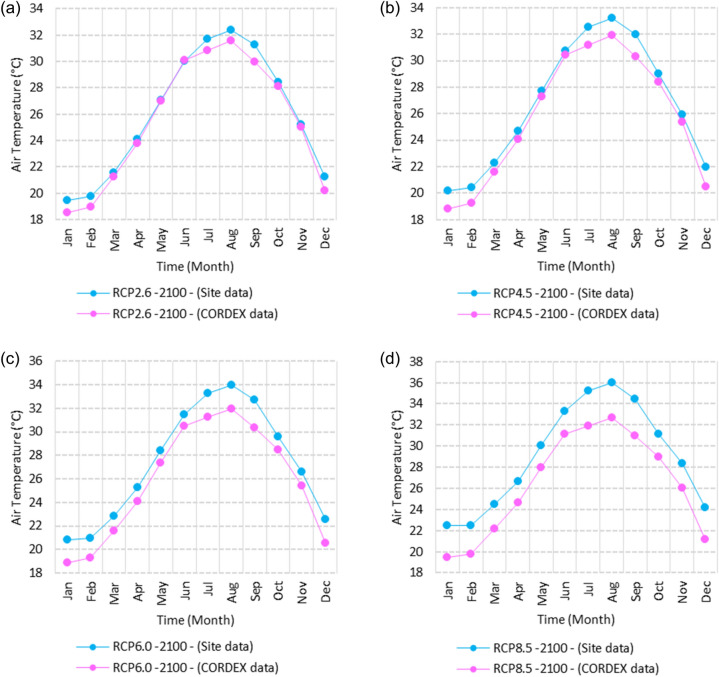
Averages of monthly maximum air temperatures according to the RCPs at Baltim. These graphs were created using Excel in the Microsoft Office Professional Plus 2016 package.

**Figure 30:**
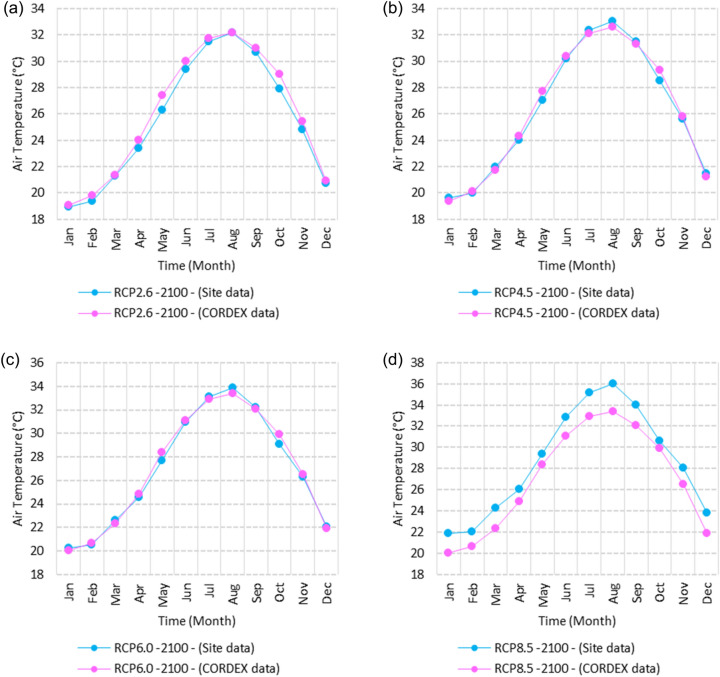
Averages of monthly maximum air temperatures according to the RCPs at Port Said. These graphs were created using Excel in the Microsoft Office Professional Plus 2016 package.

**Figure 31:**
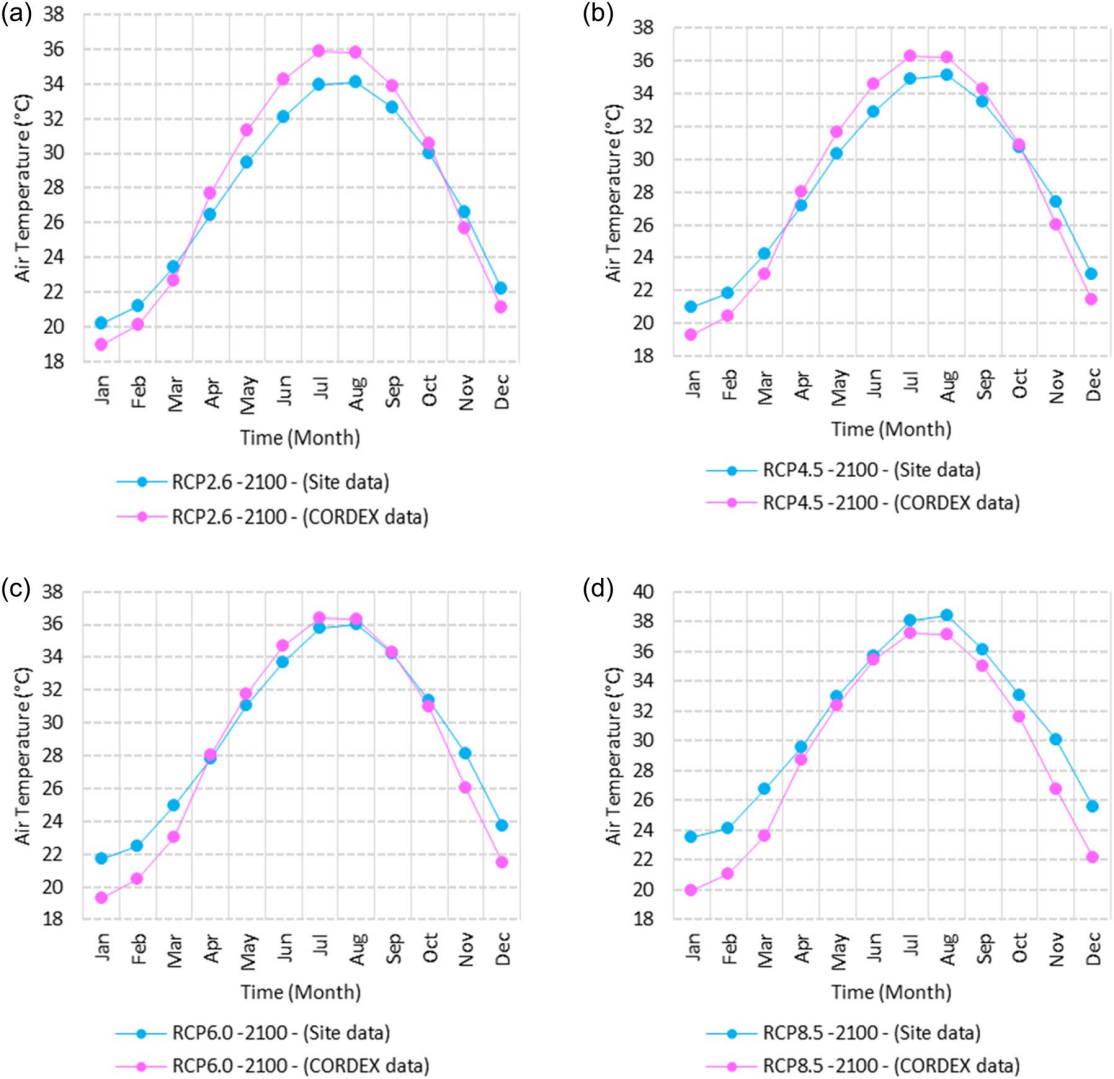
Averages of monthly maximum air temperatures according to the RCPs At al. Arish. These graphs were created using Excel in the Microsoft Office Professional Plus 2016 package.

**Figure 32:**
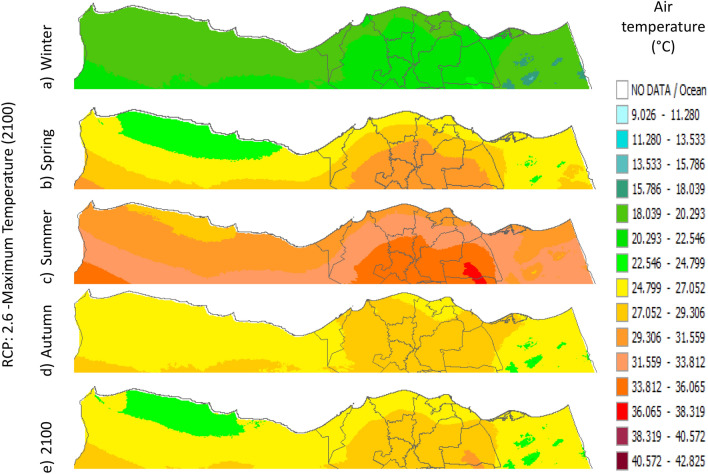
Distributions of the Projected seasonal and annual maximum air temperatures during 2100, according to RCP 2.6, along the Egyptian Mediterranean coast. The illustration was generated using SimCLIM v4.x for Desktop (SimCLIM AR5 (climsystems.com)).

**Figure 33:**
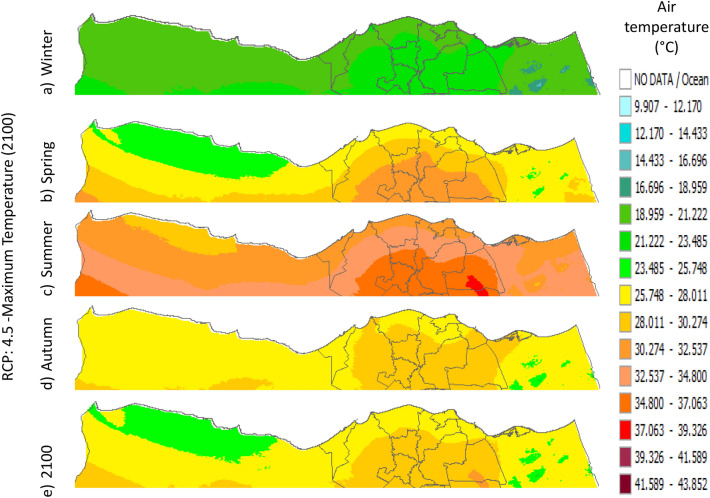
Distributions of the Projected seasonal and annual maximum air temperatures during 2100, according to RCP 4.5, along the Egyptian Mediterranean coast. The illustration was generated using SimCLIM v4.x for Desktop (SimCLIM AR5 (climsystems.com)).

**Figure 34:**
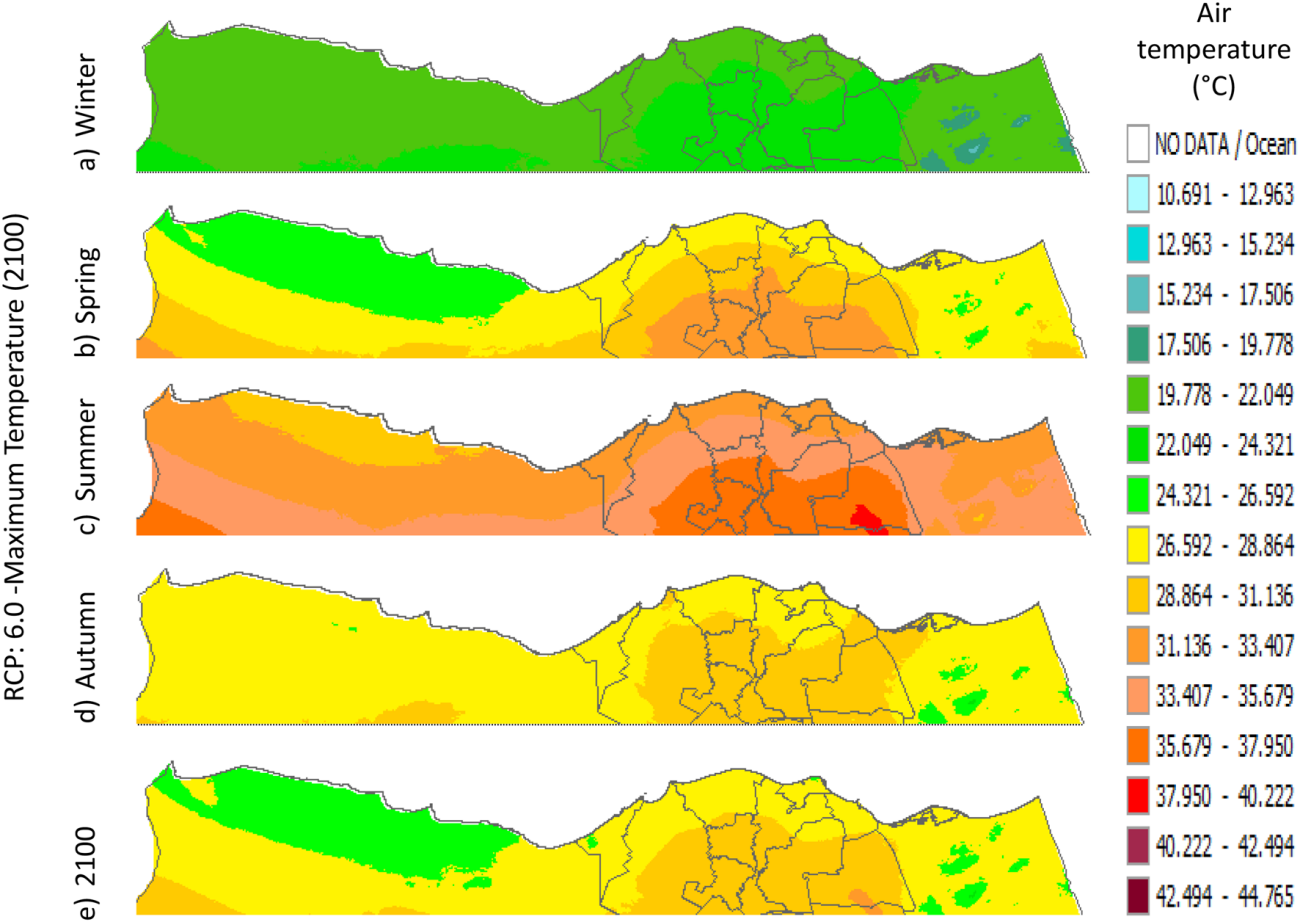
Distributions of the Projected seasonal and annual maximum air temperatures during 2100, according to RCP 6.0, along the Egyptian Mediterranean coast. The illustration was generated using SimCLIM v4.x for Desktop (SimCLIM AR5 (climsystems.com)).

**Figure 35:**
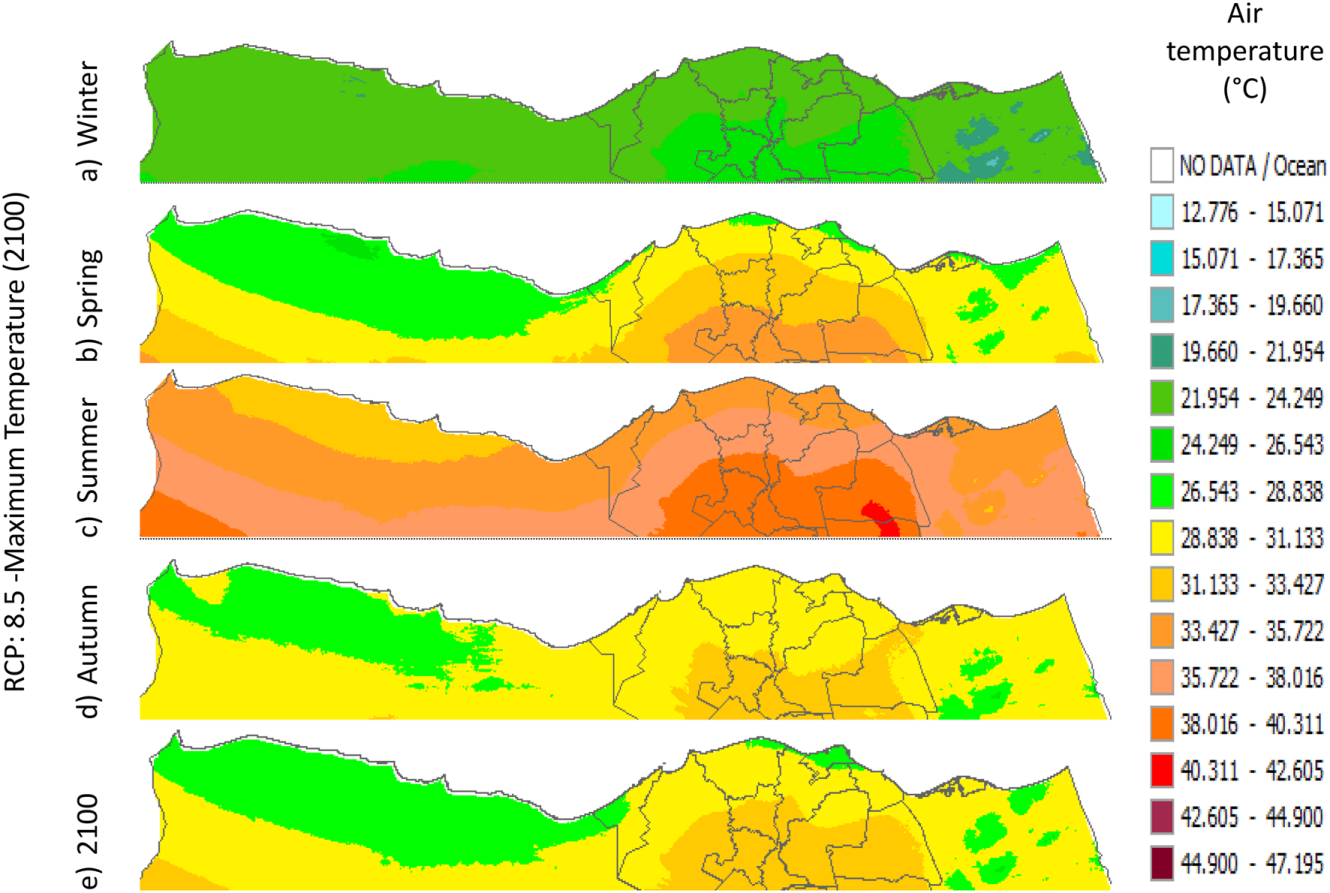
Distributions of the Projected seasonal and annual maximum air temperatures during 2100, according to RCP 8.5, along the Egyptian Mediterranean coast. The illustration was generated using SimCLIM v4.x for Desktop (SimCLIM AR5 (climsystems.com)).

### Spatial distribution of air temperature

The spatial distribution of air temperature data was examined using historical data from seven geographical positions, representing the air temperature distribution from west to east along the Egyptian Mediterranean Sea during the study's base period (2000–2016). Monthly averages of Tmin, Tmean, and Tmax were selected as fundamental descriptive statistics to simplify the results (see Fig. 3). It is evident that during the winter months (December, January, February, and March), the lowest minimum temperatures are recorded at the Al Arish station, representing the North Sinai coastal region, ranging between 7.3 and 10.02 °C (Fig. 2a). Conversely, the adjacent coastal sector (the Nile Delta) experiences the highest minimum temperatures, particularly at Port Said and Baltim stations, where temperatures range from 14.4 to 10.5 °C (Fig. 2a). The highest maximum temperature during winter was observed at the Al Arish coastal station, reaching 23.1 °C (Fig. 2b). The average winter temperature varies from 11.37 in Alexandria to 17.29 °C in Port Said. Notably, temperatures increase in Alexandria when moving east and west from the middle sector (Fig. 2c). January stands out as the coldest month of the winter season overall. In the summer, maximum temperatures at the Al Arish station peaked at 33.1 °C, especially in August, followed by Sallum station in July, reaching up to 31.5 °C (Fig. 2b). These findings align with previous studies by Domroes and El-Tantawi^9^, Hasanean and Abdel Basset^21^, Kareem et al.^14^, and and Shaltout et al.^12,13^.

### Forecasting of air temperature

Figures 2, 3, 4, 5, 6, 7, 8, 9, 10, 11, 12, 13, 14, 15, 16, 17, 18, 19, 20, 21, 22, 23, 24, 25, 26, 27, 28, 29, 30, 31, 32, 33, 34, 35 illustrate the monthly averages of Tmin, Tmean, and Tmax for both the base period and the projected averages of Tmin, Tmean, Tmax, and Tmax downscaled from 40 GCMs. Additionally, they depict the distributions of projected seasonal and annual data for the year 2100 under various Representative Concentration Pathways (RCPs).

#### Minimum temperature

Relative to the base period (2000–2016) shown in Fig. 3a at Sallum, the average Tmin was 7.3:10.02 °C and 18:22 °C during winter and summer, respectively. RCP2.6 recorded 8.6: 10.86 °C and 19.77: 22.47 °C from simulation and 7.7: 10.2 °C and 18.9: 21.1 °C during winter and summer, respectively, by projection (Fig. 3a). RCP4.5 recorded 9.3:11.53 °C and 20.6:23.38 °C by simulation and 8.07:10.5 °C and 19.2:21.5 °C during winter and summer, respectively, by projection (Fig. 3b). RCP6.0 recorded 9.98: 12.13 °C and 21.3: 24.19 °C by simulation and 8.1: 10.6 °C and 19.3: 21.6 °C during winter and summer, respectively, by projection (Fig. 3c). RCP8.5 recorded 11.6:13.72 °C and 23.29:26.34 °C from simulation and 8.7:11.5 °C and 20:22.04 °C during winter and summer, respectively, by projection (Fig. 3d).

Relative to the base period (2000–2016) shown in Fig. 4a at Mersah Matruh, the average Tmin was 9.5:11:2 °C and 19.4:22.7 °C during winter and summer, respectively. RCP2.6 recorded 10.3:11.88 °C and 20.05:23.69 °C from simulation and 8.9:11.1 °C and 20.42:23.1 °C during winter and summer, respectively, by projection (Fig. 4a). RCP4.5 recorded 11.11:12.68 °C and 21.2:24.45 °C with simulation, and 9.3:11.4 °C and 20.4:23.5 °C with projection during winter and summer, respectively (Fig. 4b). RCP6.0 recorded 11.6:13.3 °C and 21.8:25.3° by simulation and 9.34:11.46 °C and 20.4:23.5 °C during winter and summer, respectively, by projection (Fig. 4c). RCP8.5 recorded 13.28:14.65 °C and 23.7:27.38 °C from simulation and 9.9:12.02 °C and 21.1:24.3 °C during winter and summer, respectively, by projection (Fig. 4d).

Relative to the base period (2000–2016) shown in Fig. 5a at Dabaa, the average Tmin was 9.4:11.6 °C and 20.2:23.1 °C during winter and summer, respectively. RCP2.6 recorded 10.37:12.49 °C and 21.2:24.3 °C using simulation and 8.1:10.25 °C and 19.3:22.1 °C during winter and summer, respectively, using projection (Fig. 5a). RCP4.5 recorded 11.1: 13.17 °C and 22: 25.12 °C using simulation, and 8.4: 10.5 °C and 19.7: 22.5 °C during winter and summer, respectively, by projection (Fig. 5b). RCP6.0 recorded 11.77:13.7 °C and 22.7:25.9 °C using simulation and 8.5:10.6 °C and 19.7:22.6 °C during winter and summer, respectively, by projection (Fig. 5c). RCP8.5 recorded 13.5:15.53 °C and 24.5:27.9 °C using simulation and 9.1:11.3 °C and 20.4:23.3 °C during winter and summer, respectively, by projection (Fig. 5d).

Relative to the base period (2000–2016) shown in Fig. 6a at Alexandria, the average Tmin was 9.2:11.6 °C and 21.4:24.3 °C during winter and summer, respectively. RCP2.6 recorded 10.05:12.15 °C and 22.36:25.3 °C using simulation and 10.35:12.27 °C and 21.3:22.57 °C, respectively, during winter and summer by projection (Fig. 6a). RCP4.5 recorded 10.82:12.82 °C and 23.18:26.2 °C using simulation and 10.69:12.62 °C and 21.6:23.9 °C during winter and summer, respectively, by projection (Fig. 6b). RCP6.0 recorded 11.5: 13.49 °C and 23.9: 27.1 °C using simulation and 10.7: 12.67 °C and 21.7: 24 °C, respectively, during winter and summer by projection (Fig. 6c). RCP8.5 recorded 13:15 °C and 25:89 °C using simulation and 11.39:11.34 °C and 22.4:24.8 °C during winter and summer, respectively, by projection (Fig. 6d).

Relative to the base period (2000–2016) shown in Fig. 7a at Baltim, the average Tmin was 10.56:12.87 °C and 21.7:24.6 °C during winter and summer, respectively. RCP2.6 recorded 11.48: 13.38 °C and 22.52: 25.44 °C using simulation and 11.8: 13.13 °C and 21.2: 24.23 °C during winter and summer, respectively, by projection (Fig. 7a). RCP4.5 recorded 12.2:14.09 °C and 23.2:26.2 °C using simulation and 11.8:13.46 °C and 21.5:24.6 °C during winter and summer, respectively, by projection (Fig. 7b). RCP6.0 recorded 12.84:14.76 °C and 23.9:27.05 °C using simulation and 11.8:13.5 °C and 21.6:24.6 °C during winter and summer, respectively, by projection (Fig. 7c). RCP8.5 recorded 14.49:16.53 °C and 25.7:29 °C using simulation and 13.9:14.1 °C and 22.26:25.39 °C during winter and summer, respectively, by projection (Fig. 7d).

Relative to the base period (2000–2016) shown in Fig. 8a at Port Said, the average Tmin was 11.74:14.48 °C and 23:25.7 °C during winter and summer, respectively. RCP2.6 recorded 12.85:15.15 °C and 39.9:26.8 °C using simulation and 11.4:13.6 °C and 22.25:24.7 °C during winter and summer, respectively, by projection (Fig. 8a). RCP4.5 recorded 13.57:15.84 °C and 24.7:27.7 °C using simulation, and 11.8:13.9 °C and 22.5:25.1 °C during winter and summer, respectively, by projection (Fig. 8b). RCP6.0 recorded 14.21:16.44 °C and 25.4:28.5 °C using simulation and 11.8:14 °C and 22.6:25.2 °C during winter and summer, respectively, by projection (Fig. 8c). RCP8.5 recorded 15.9:18.06 °C and 27:2:30.65 °C using simulation and 12.47:14.6 °C and 23.2:26 °C during winter and summer, respectively, by projection (Fig. 8d).

Relative to the base period (2000–2016) shown in Fig. 9a at Al Arish, the average Tmin was 8.1:10.2 °C and 18.6:22 °C during winter and summer, respectively. RCP2.6 recorded 8.28:10.62 °C and 19.7:23.07 °C using simulation and 9.52:11.87 °C and 21.5:24.24 °C during winter and summer, respectively, by projection (Fig. 9a). RCP 4.5 recorded 8.98:11.33 °C and 20.56:24.03 °C using simulation and 9.8:12.17 °C and 21.84:24.68 °C during winter and summer, respectively, by projection (Fig. 9b). RCP 6.0 recorded 9.6: 11.9 °C and 21.33: 24.89 °C using simulation and 8.8: 11.24 °C and 20.75: 23.32 °C during winter and summer, respectively, by projection (Fig. 9c). RCP8.5 recorded 11.28:12.72 °C and 23.63:27.18 °C using simulation and 10.46:12.77 °C and 22.54:25.56 °C during winter and summer, respectively, by projection (Fig. 9d).

The seasonal distribution of the Projected average Tmin according to RCP 2.6 from the west to the east along the Egyptian Mediterranean coastal area during winter, spring, summer, and autumn will have an average of 5.9:11.8 °C, 13.75:17.63 °C, 19.57:23.44 °C, and 15.6:17.6 °C, respectively (Fig. 10). RCP 4.5 recorded average Tmin of 8.84:12.75 °C, 14.7:18.6 °C, 20.5:24.4 °C, and 16.6:20.5 °C, respectively (Fig. 11). RCP 6.0 recorded average Tmin of 7.6:13.5 °C, 15.5:19.5 °C, 21.4:25.4 °C, and 15.5:19.5 °C, respectively (Fig. 12). RCP 8.5 recorded average Tmin of 11.8:15.8 °C, 15.8:19.8 °C, 23.8:27.8 °C, and 17.8:21.8 °C, respectively (Fig. 13).

#### Mean temperature

Relative to the base period (2000–2016) shown in Fig. 2b at Sallum, the average Tmean was 12.1:15.1 °C and 23.7:26.1 °C during winter and summer, respectively. RCP2.6 recorded 12.9: 15.8 °C and 24.8:27.3 °C from simulation and 12.6:15.4 °C and 24.06:25.6 °C during winter and summer, respectively, by projection (Fig. 14a). RCP4.5 recorded 13.6:16.5 °C and 25.6:28.2 °C by simulation and 12.9:15.7 °C and 24.4:26 °C during winter and summer, respectively, by projection (Fig. 14b). RCP6.0 recorded 14.2: 17.1 °C and 26.3: 29.09 °C by simulation and 13:15.8 °C and 24.4: 26 °C during winter and summer, respectively, by projection (Fig. 14c). RCP8.5 recorded 15.83:18.76 °C and 28.2:31.31 °C from simulation and 13.5:16.3 °C and 25.1:26.8 °C during winter and summer, respectively, by projection (Fig. 14d).

Relative to the base period (2000–2016) shown in Fig. 2b at Mersah Matruh, the average Tmean was 14.2:16 °C and 24:26.8 °C during winter and summer, respectively. RCP2.6 recorded 14.49:16.6 °C and 25:27.8 °C from simulation and 14.1:15.9 °C and 23.7:26.1 °C during winter and summer, respectively, by projection (Fig. 15a). RCP4.5 recorded 15.1:17.26 °C and 25.76:28.76 °C with simulation, and 13.9:16.2 °C and 24:26.5 °C with projection during winter and summer, respectively (Fig. 15b). RCP6.0 recorded 15.7:17.8 °C and 26.4:29.5° by simulation and 14.1:16.1 °C and 24.1:26.6 °C during winter and summer, respectively, by projection (Fig. 15c). RCP8.5 recorded 13.38:19.39 °C and 28.24:30.74 °C from simulation and 14.5:16.8 °C and 24.7:27.3 °C during winter and summer, respectively, by projection (Fig. 15d).

Relative to the base period (2000–2016) shown in Fig. 2b at Dabaa, the average Tmean was 13.7:16.4 °C and 24.5:27.4 °C during winter and summer, respectively. RCP2.6 recorded 14.64:17.1 °C and 25.49:28.47 °C using simulation and 13.4:15.4 °C and 23.8:26.2 °C during winter and summer, respectively, using projection (Fig. 16a). RCP4.5 recorded 15.36: 17.8 °C and 26.27: 29.36 °C using simulation, and 13.7: 15.7 °C and 24.2: 26.9 °C during winter and summer, respectively, by projection (Fig. 16b). RCP6.0 recorded 15.9:18.4 °C and 26.9:30.1 °C using simulation and 13.76:15.78 °C and 24.2:26.7 °C during winter and summer, respectively, by projection (Fig. 16c). RCP8.5 recorded 17.6:20 °C and 28.8:32.24 °C using simulation and 14.37:16.35 °C and 24.9:27.4 °C during winter and summer, respectively, by projection (Fig. 16d).

Relative to the base period (2000–2016) shown in Fig. 2b at Alexandria, the average Tmean was 11.3:16.6 °C and 25.2:27.8 °C during winter and summer, respectively. RCP2.6 recorded 14.7:17.1 °C and 26.1:28.7 °C using simulation and 14.4:16.8 °C and 25.5:27.6 °C, respectively, during winter and summer by projection (Fig. 17a). RCP4.5 recorded 15.53:17.84 °C and 26.97:29.64 °C using simulation and 14.7:17.1 °C and 25.95:28.05 °C during winter and summer, respectively, by projection (Fig. 17b). RCP6.0 recorded 16.2: 18.44 °C and 27.7: 30.4 °C using simulation and 14.8: 17.2 °C and 26: 28 °C, respectively, during winter and summer by projection (Fig. 17c). RCP8.5 recorded 13:15 °C and 25:89 °C using simulation and 18.1:20.05 °C and 29.6:32.5 °C during winter and summer, respectively, by projection (Fig. 17d).

Relative to the base period (2000–2016) shown in Fig. 2b at Baltim, the average Tmean was 14.1:16.6 °C and 25.1:27.8 °C during winter and summer, respectively. RCP2.6 recorded 15.7: 17.1 °C and 25.96: 28.63 °C using simulation and 15: 17 °C and 25.6: 27.8 °C during winter and summer, respectively, by projection (Fig. 18a). RCP4.5 recorded 15.8:17.8 °C and 26.7:29.49 °C using simulation and 15.43:17.45 °C and 25.9:28.2 °C during winter and summer, respectively, by projection (Fig. 18b). RCP6.0 recorded 16.45:18.43 °C and 27.38:30.25 °C using simulation and 15.5:17.4 °C and 26:28.2 °C during winter and summer, respectively, by projection (Fig. 18c). RCP8.5 recorded 18.17:20.1 °C and 29.16:32.28 °C using simulation and 16:18 °C and 26.65:29 °C during winter and summer, respectively, by projection (Fig. 18d).

Relative to the base period (2000–2016) shown in Fig. 2b at Port Said, the average Tmean was 14.7:17.2 °C and 25.5:28.2 °C during winter and summer, respectively. RCP2.6 recorded 15.6:17.8 °C and 26.3:29.1 °C using simulation and 15.3:17.4 °C and 26:28.4 °C during winter and summer, respectively, by projection (Fig. 19a). RCP4.5 recorded 16.4:18.5 °C and 27:30 °C using simulation, and 15.6:17.7 °C and 26.4:28.8 °C during winter and summer, respectively, by projection (Fig. 19b). RCP6.0 recorded 17:19 °C and 27.86:30.83 °C using simulation and 15.7:17.8 °C and 26.4:28.9 °C during winter and summer, respectively, by projection (Fig. 19c). RCP8.5 recorded 18.82:20.72 °C and 29.7:32.9 °C using simulation and 16.7:18.3 °C and 27.1:29.65 °C during winter and summer, respectively, by projection (Fig. 19d).

Relative to the base period (2000–2016) shown in Fig. 2b at Al Arish, the average Tmean was 12.7:16.2 °C and 24.9:27.5 °C during winter and summer, respectively. RCP2.6 recorded 13.94:16.93 °C and 25.85:28.48 °C using simulation and 12.7:15.8 °C and 24.3:27 °C during winter and summer, respectively, by projection (Fig. 20a). RCP 4.5 recorded 14.7:17.6 °C and 26.69:29.45 °C using simulation and 13:16.15 °C and 24.6:27.4 °C during winter and summer, respectively, by projection (Fig. 20b). RCP 6.0 recorded 15.39: 18.33 °C and 27.44: 30.31 °C using simulation and 13.1: 16.2 °C and 24.7: 27.5 °C during winter and summer, respectively, by projection (Fig. 20c). RCP8.5 recorded 17.2:20 °C and 29.42:32.6 °C using simulation and 13.8:16.8 °C and 25.46:28.3 °C during winter and summer, respectively, by projection (Fig. 20d).

The seasonal distribution of the Projected average Tmean according to RCP 2.6 from the west to the east along the Egyptian Mediterranean coastal area during winter, spring, summer, and autumn will have an average of 14:16.18 °C, 18.2:22.4 °C, 24.5:28.7 °C, and 20.38:24.57 °C, respectively (Fig. 21). RCP 4.5 recorded average Tmean of 15:19.23 °C, 19.2:23.4 °C, 25.5:29.7 °C, and 21.3:23.4 °C, respectively (Fig. 22). RCP 6.0 recorded average Tmean of 15.8:20 °C, 20:24.32 °C, 26.4:30.6 °C, and 20:24.32 °C, respectively (Fig. 23). RCP 8.5 recorded average Tmean of 15.8:20.19 °C, 20:24 °C, 26.6:30.9 °C, and 22.3:26.6 °C, respectively (Fig. 24).

#### Maximum temperature

Relative to the base period (2000–2016) that shows in Fig. 2c at Sallum, the average Tmax was 17.05:20.5 °C and 29.29:31.58 °C during winter and summer, respectively. RCP2.6 recorded 17.97: 21.24 °C and 30.25:32.74 °C from simulation and 17.65:20.68 °C and 29:30.4 °C during winter and summer, respectively, by projection (Fig. 25a). RCP4.5 recorded 18.64:21.94 °C and 31.04:33.72 °C by simulation and 17.9:20.9 °C and 29.3:30.8 °C during winter and summer, respectively, by projection (Fig. 25b). RCP6.0 recorded 19.24: 22.56 °C and 31.74: 34.58 °C by simulation and 17.9:21 °C and 29.4:30.9 °C during winter and summer, respectively, by projection (Fig. 25c). RCP8.5 recorded 20.82:24.22 °C and 33.6:36.9 °C from simulation and 18.5:21.6 °C and 30:31.6 °C during winter and summer, respectively, by projection (Fig. 25d).

Relative to the base period (2000–2016) shown in Fig. 2c at Mersah Matruh, the average Tmax was 18.2:20.94 °C and 28.19:30.67 °C during winter and summer, respectively. RCP2.6 recorded 19.07:21.43 °C and 29.19:31.66 °C from simulation and 18.4:20.7 °C and 27.66:29.28 °C during winter and summer, respectively, by projection (Fig. 26a). RCP4.5 recorded 19.72:22.11 °C and 29.97:32.56 °C with simulation, and 18.7:21.06 °C and 28:29.68 °C with projection during winter and summer, respectively (Fig. 26b). RCP6.0 recorded 20.3:22.7 °C and 30.66:33.37° by simulation and 18.77:21.1 °C and 28:29.7 °C during winter and summer, respectively, by projection (Fig. 26c). RCP8.5 recorded 21.85:24.3 °C and 32.5:35.5 °C from simulation and 19.3:21.68 °C and 28.:30.5 °C during winter and summer, respectively, by projection (Fig. 26d).Relative to the base period (2000–2016) shown in Fig. 2c at Dabaa, the average Tmax was 18.89:21.4 °C and 29:31.5 °C during winter and summer, respectively. RCP2.6 recorded 19.72:22.29 °C and 30:32.65 °C using simulation and 18.6:20.7 °C and 28.3:30.39 °C during winter and summer, respectively, using projection (Fig. 27a). RCP4.5 recorded 20.41:22.99 °C and 30.8: 33.65 °C using simulation, and 18.9: 21.09 °C and 28.74: 30.8 °C during winter and summer, respectively, by projection (Fig. 27b). RCP6.0 recorded 21.3:23.6 °C and 31.5:34.38 °C using simulation and 19:21 °C and 28.79:30.86 °C during winter and summer, respectively, by projection (Fig. 27c). RCP8.5 recorded 22.68:25.26 °C and 33.39:36.55 °C using simulation and 19.6:21.7 °C and 29.47:31.65 °C during winter and summer, respectively, by projection (Fig. 27d).

Relative to the base period (2000–2016) shown in Fig. 2c at Alexandria, the average Tmax was 18.43:21.6 °C and 29.28:31.34 °C during winter and summer, respectively. RCP2.6 recorded 19.43:22.12 °C and 30.22:32.34 °C using simulation and 18.61:21.5 °C and 30:31.8 °C, respectively, during winter and summer by projection (Fig. 28a). RCP4.5 recorded 20.18:22.84 °C and 31:33.3 °C using simulation and 18.9:21.8 °C and 30.3:32.2 °C during winter and summer, respectively, by projection (Fig. 28b). RCP6.0 recorded 20.85: 23.48 °C and 31.79: 34.15 °C using simulation and 19: 21.86 °C and 30.43: 32.32 °C, respectively, during winter and summer by projection (Fig. 28c). RCP8.5 recorded 22.63:25.18 °C and 33.57:36.42 °C using simulation and 19.65:22.74 °C and 31.14:33.14 °C during winter and summer, respectively, by projection (Fig. 28d).

Relative to the base period (2000–2016) shown in Fig. 2c at Baltim, the average Tmax was 18.47:21 °C and 29.15:31.58 °C during winter and summer, respectively. RCP2.6 recorded 19.46: 21.57 °C and 30:32.35C using simulation and 18.4: 21.2 °C and 30: 31.5 °C during winter and summer, respectively, by projection (Fig. 29a). RCP4.5 recorded 20.18:22.26 °C and 30.77:32.55 °C using simulation and 18.81:21.57 °C and 30.4:31.9 °C during winter and summer, respectively, by projection (Fig. 29b). RCP6.0 recorded 20.8:22.87 °C and 31.47:33.97 °C using simulation and 18.8:21.6 °C and 30.32:31.9 °C during winter and summer, respectively, by projection (Fig. 29c). RCP8.5 recorded 22.2:24.48 °C and 33.31:36 °C using simulation and 19.4:22.2 °C and 30.9:32.7 °C during winter and summer, respectively, by projection (Fig. 29d).

Relative to the base period (2000–2016) shown in Fig. 2c at Port Said, the average Tmax was 18.18:20.75 °C and 28.59:31.2 °C during winter and summer, respectively. RCP2.6 recorded 18.93:21.28 °C and 29.39:32.16 °C using simulation and 19:21.3 °C and 29.9:32.18 °C during winter and summer, respectively, by projection (Fig. 30a). RCP4.5 recorded 19.63:21.98 °C and 30.21:33 °C using simulation, and 19.3:21.6 °C and 30.3:32.5 °C during winter and summer, respectively, by projection (Fig. 30b). RCP6.0 recorded 20.25:22.6 °C and 30.9:33.87 °C using simulation and 20:22.3 °C and 31.1:33.4 °C during winter and summer, respectively, by projection (Fig. 30c). RCP8.5 recorded 21.9:24.25 °C and 32.86:36 °C using simulation and 20:22.3 °C and 31:33.4 °C during winter and summer, respectively, by projection (Fig. 30d). Relative to the base period (2000–2016) shown in Fig. 2c at Al Arish, the average Tmax was 19.29:23 °C and 31.2:33.26 °C during winter and summer, respectively. RCP2.6 recorded 20.19:23.44 °C and 32:34.13 °C using simulation and 19.8:22.6 °C and 33.8:35.8 °C during winter and summer, respectively, by projection (Fig. 31a). RCP 4.5 recorded 20.9:24.22 °C and 32.92:35.13 °C using simulation and 19.3:22.9 °C and 34.2:36.3 °C during winter and summer, respectively, by projection (Fig. 31b). RCP 6.0 recorded 21.68: 24.92 °C and 33.68: 36 °C using simulation and 19.3: 23 °C and 34.3: 36.37 °C during winter and summer, respectively, by projection (Fig. 31c). RCP8.5 recorded 23.53:26.78 °C and 35.69:38.37 °C using simulation and 19.9:23.6 °C and 35:37.2 °C during winter and summer, respectively, by projection (Fig. 31d).

#### The change between the base period and 2100

**Figure 36:**
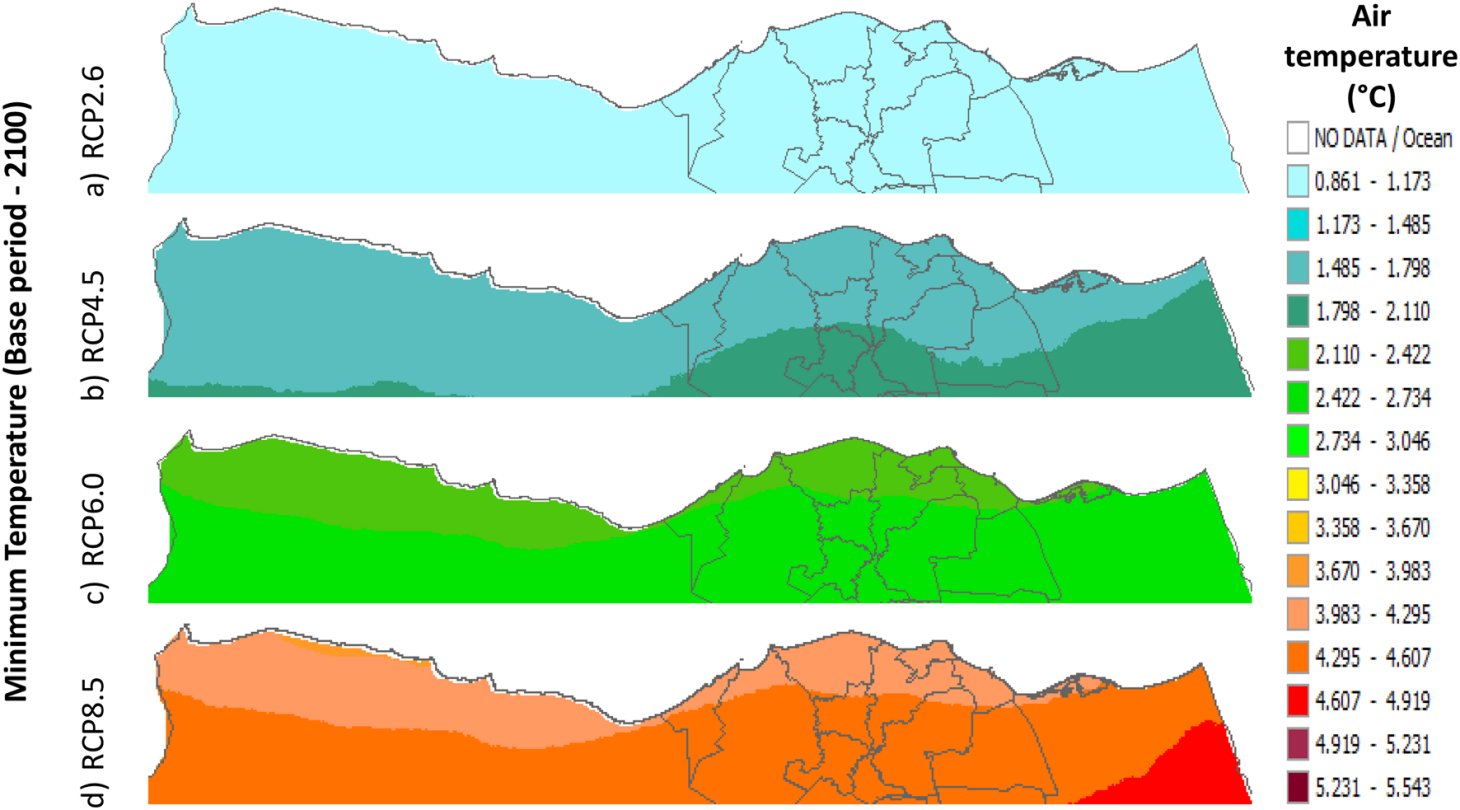
The change in average minimum temperature between the base period and 2100, according to RCPs, along the Egyptian Mediterranean coast. The illustration was generated using SimCLIM v4.x for Desktop (SimCLIM AR5 (climsystems.com)).

**Figure 37:**
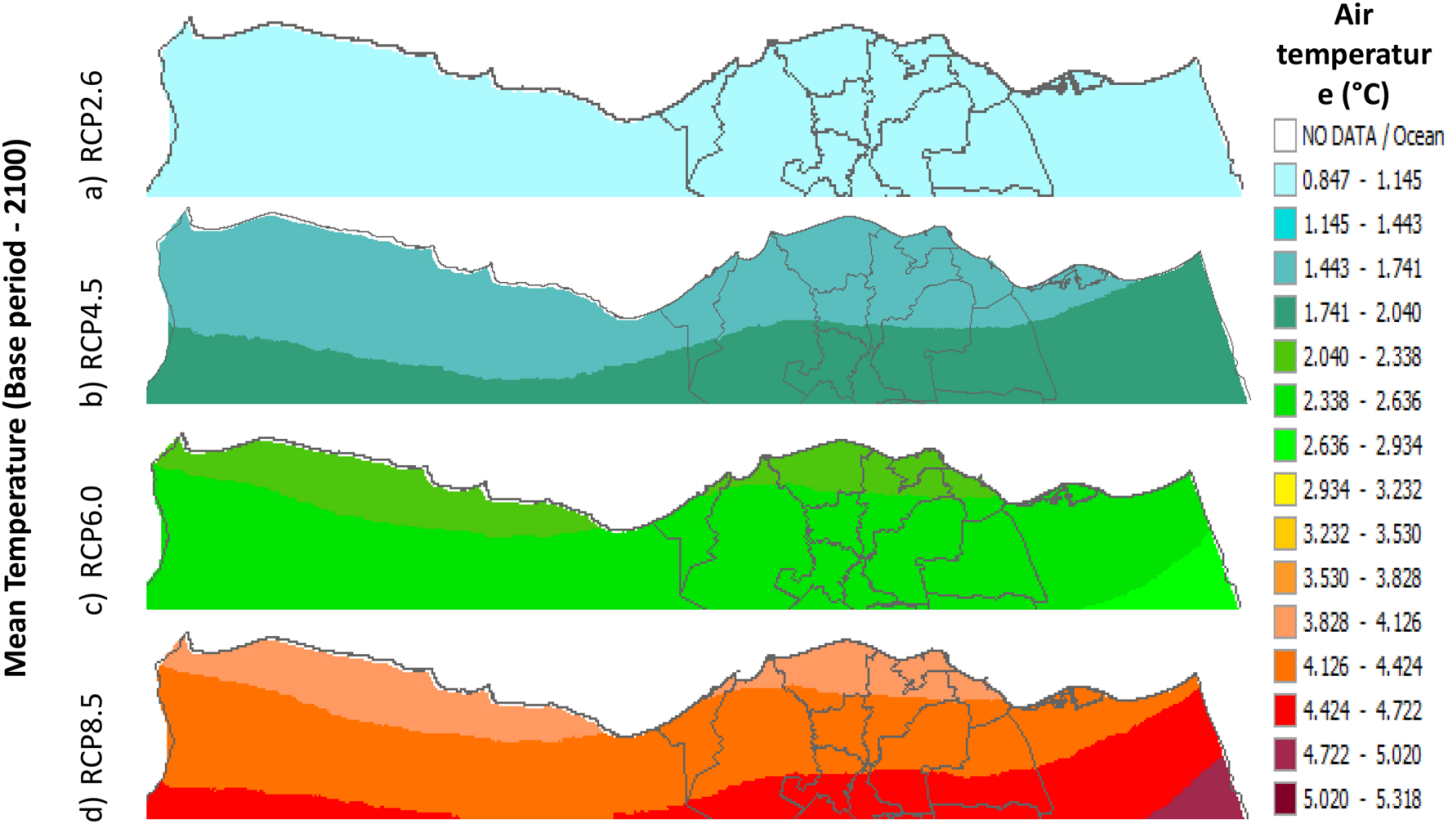
The Change in average mean temperature between the base period and 2100, according to RCPs, along the Egyptian Mediterranean coast. The illustration was generated using SimCLIM v4.x for Desktop (SimCLIM AR5 (climsystems.com)).

**Figure 38:**
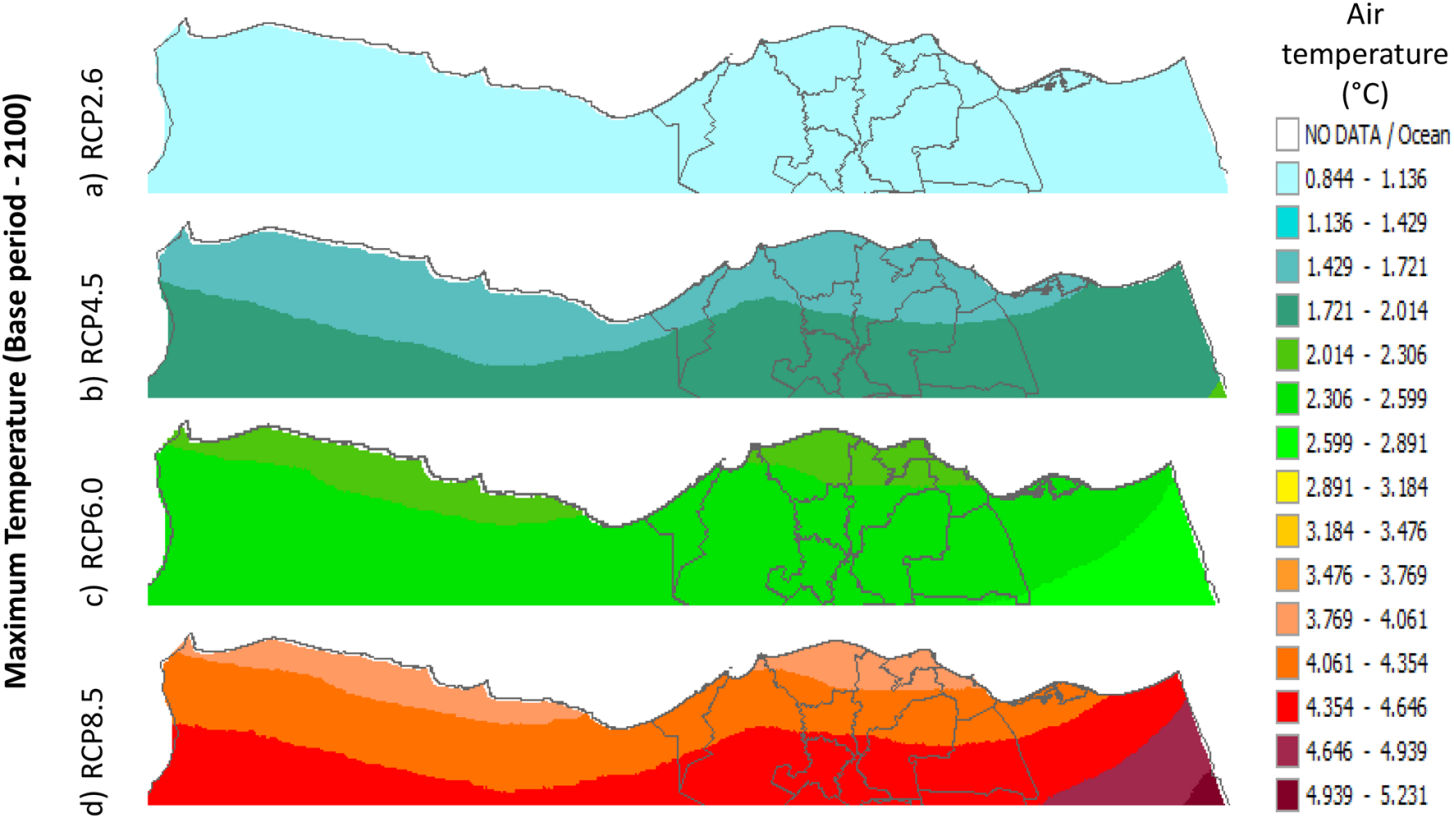
The Change in average Maximum temperature between the base period and 2100, according to RCPs, along the Egyptian Mediterranean coast. The illustration was generated using SimCLIM v4.x for Desktop (SimCLIM AR5 (climsystems.com)).

Figures (36:38) show the estimation limits of the minimum, mean, and maximum temperature increase output from the middle climate sensitivity with respect to the base period and, according to RCPs 2.6, 4.5, 6.0, and 8.5 during 2100, downscaled from 74 pattern ensembles, are from west to east along the Egyptian Mediterranean coast. Relative to Tmin, they are as follows: 0.86:1.173 °C, 1.48:1.79 °C, 2.1:2.7 °C, and 3.9:4.2 °C, respectively. Also, relative to the Tmean, they are as follows: 0.8:1 °C, 1.4:2 °C, 2:2.6 °C, and 3.8:4.4 °C, respectively. Further, relative to Tmax, they are as follows: 0.8:1 °C, 1.7:2 °C, 2.3:3.8 °C, and 3.7:4.6 °C, respectively (Figs. 36, 37, 38).

#### Results of SimCLIM evaluation

Results of the SimCLIM evaluation reveal that at the Port Said station, in relation to RCPs 2.6 and 8.5 for 2040, the Projected Tmean using climate pattern ensembles will be 22.54  °C and 23.024  °C, respectively. The downscaled Tmean values are projected to be 22.093 °C and 22.567 °C, respectively. The percentage prediction error range between the Projected and downscaled Tmean is approximately 2%.

## Discussion and conclusions

The SimCLIM model combined with 40 GCMs and 74 climate pattern ensembles collaborated by IPCC AR5 GHG emission scenarios predicts a dominant upward trend in air temperature for the year 2100 in the Egyptian Mediterranean coastal area, which is extremely compatible with previous studies concerned with temperature forecasting either at the global scale (Zittis^22^) or at the regional scale (Nastos and Kapsomenakis^23^, Philandras et al.^24^, ^25^) where all agreed that there would be an upward tendency in the future relative to the air temperature. Furthermore, the positive temperature inclination projected in this research supports the temperature projection findings in the area linked to earlier studies (Elbessa et al.^26^). The results show that, along the Egyptian Mediterranean coast during the sixteen years (2000–2016) using (GHCN) data, the average air temperatures varied from west to east during the winter months (December–March) between 17.29 °C recorded at Port Said and 11.3 °C recorded at Alexandria, with the lowest average mean in January's months. Further, during the summer, the Tmean peaked at Port Said, where averages of 28.23 °C recorded the highest average air temperature. During the spring months (April and May), the temperature varied from 17.6  °C at Sallum to 21.9 °C at Port Said. Also, during the autumn (October and November), the temperature varied from 17.58 °C at Sallum to 24.9 °C at Port Said, which shows an increase in the mean temperature from west to east. Relative to RCP 8.5, the increase in temperature will be up to 3.9 °C to 4.6 °C by 2100. Moreover, there were ups and downs in Tmin and Tmax in between sectors, but a general increase trend from west to west was confirmed by (Elbessa et al.^27^) using the RegCM-SVN model. This model is highly recommended in the evaluation of future climate scenarios.

Global observations have noted fluctuations in extreme temperature markers^28,29^, which align with the unique temperature anomalies recorded at the Al Arish station. This station reports both the lowest minimum temperatures in winter and the highest maximum temperatures in winter. Several factors influence the climate of Al Arish, including microclimatic elements, such as its geographical location in the desert of the North Sinai Peninsula. It is just a few miles from the northern coastline and a short distance from a mountain range to the south. As a result, the area experiences specific meteorological phenomena, such as breezes, katabatic winds, and anabatic winds^26^. Additionally, this anomaly could be attributed to the influence of thermohaline circulation in the eastern Mediterranean and winter mixing typically observed in December–January. As reported by Menna et al.^30^, these factors affect the air above the sea.

## Data Availability

Correspondence and requests for materials should be addressed to N.S., K.T. or R.C.
